# Medulloblastoma in Adolescents and Young Adults: Molecular Subgroups, Prognostic Biomarkers, and Age-Specific Therapeutic Challenges

**DOI:** 10.3390/cimb48030297

**Published:** 2026-03-11

**Authors:** Antonio Ruggiero, Marco Gessi, Antonio d’Amati, Alessio Albanese, Giorgio Attinà

**Affiliations:** 1 Pediatric Oncology Unit, Fondazione Policlinico Universitario Agostino Gemelli IRCCS, 00168 Rome, Italy; giorgio.attina@policlinicogemelli.it; 2 Department of Woman and Child Health, Università Cattolica del Sacro Cuore, 00168 Rome, Italy; marco.gessi@policlinicogemelli.it (M.G.); antonio.damati@guest.policlinicogemelli.it (A.d.); 3 Pathology Unit, Fondazione Policlinico Universitario A. Gemelli-IRCCS, 00168 Rome, Italy; 4 Neurosurgery Unit, Department of Neurosciences, Fondazione Policlinico Universitario Agostino Gemelli IRCCS, 00168 Rome, Italy; alessio.albanese@unicatt.it; 5 Neurosurgery Unit, Department of Neurosciences, Università Cattolica del Sacro Cuore, 00168 Rome, Italy

**Keywords:** medulloblastoma, adolescent and young adult, molecular subgroups, TP53 mutations, MYCN amplification, prognosis, targeted therapy

## Abstract

Medulloblastoma is the most common malignant brain tumor in children, but it presents distinct challenges when occurring in adolescents and young adults (AYAs, aged 15–39 years). Recent molecular profiling has identified four principal medulloblastoma subgroups—WNT-activated, SHH-activated, Group 3, and Group 4—each demonstrating unique biological characteristics and clinical outcomes. AYA patients exhibit age-specific molecular patterns and therapeutic responses substantially different from those of younger children. This review synthesizes current evidence regarding epidemiology, diagnostic challenges, molecular characterization, risk stratification, treatment modalities, and outcomes specific to AYA medulloblastoma patients, highlighting the critical need for age-adapted therapeutic strategies and dedicated clinical research in this underserved population.

## 1. Introduction

Medulloblastoma accounts for about 20–25% of brain tumors in children and accounts for 63% of intracranial embryonal neoplasms [1,2]. These highly aggressive cerebellar tumors are very diverse both in terms of their molecular and clinical features. Mostly a childhood tumor, medulloblastoma is very rarely diagnosed in adolescents and young adults, in whom it constitutes less than 1–2% of central nervous system (CNS) tumors, with an annual incidence rate of 0.6–1 per million population [3,4].

The adolescent and young adult (AYA) oncology population, usually referred to as people aged 15–39 years at the time of diagnosis, holds a special place between pediatric and adult care paradigms and is faced with distinct biological, psychological, and socioeconomic challenges [5]. AYA patients with medulloblastoma, despite being a clearly defined clinical group, are still very poorly represented in clinical trials; there is a big gap in evidence-based knowledge for optimal treatment and long-term outcomes [6].

Medulloblastoma molecular classification has revolutionized the knowledge of its heterogeneity. Different studies on gene expression profiling have led to the identification of four major molecular subgroups: WNT-activated, sonic hedgehog (SHH) activated, Group 3, and Group 4, each having its distinct transcriptomic profiles, genetic abnormalities, age distributions, and clinical outcomes [7,8]. In its 2016 and 2021 editions, the WHO classifications of CNS tumors introduced molecular subgrouping into the diagnostic criteria, thus acknowledging this as an essential element for classification and risk stratification [9].

Recent studies have revealed substantial differences in the medulloblastoma biology of children and adults, implying that pediatric and adult medulloblastomas might actually be two different kinds of diseases [10,11]. SHH-activated tumors dominate in AYA patients, whereas Group 3 tumors are relatively scarce compared with the younger child group. In addition, molecular changes typical for certain ages, such as TP53 mutations, MYCN amplifications, and TERT promoter mutations, are found to have different prognostic implications when compared across different age groups [12,13].

In this thorough review, the authors focus on the current knowledge about AYA medulloblastoma, collating the evidence on AYA-specific epidemiology, molecular characterization, prognostic biomarkers, therapeutic approaches, and survivorship issues. This compilation aids comprehension. Knowledge of these age-specific features is vital for formulating logical, biologically informed treatment strategies and for enhancing positive patient outcomes in this neglected population.

## 2. Epidemiology and Clinical Presentation

### 2.1. Age Distribution and Demographics

Medulloblastoma demonstrates bimodal age distribution, with peak incidence during early childhood (3–7 years) and a smaller secondary peak in early adulthood [4,14]. In AYAs, annual incidence is approximately 0.6–1.0 per million individuals aged 15–39 years [3]. SEER database analysis of 857 adult patients diagnosed between 1973 and 2014 showed medulloblastoma most commonly presents in the 20–29 year age group, with a slight male preponderance (58.5%) and predominantly cerebellar localization (91.6%) [15].

Gender distribution varies across molecular subgroups. Group 3 and Group 4 demonstrate pronounced male predominance (approximately 2:1 male-to-female ratio) [16]. In contrast, WNT-activated tumors exhibit relatively balanced gender distribution, while SHH-activated tumors show variable gender ratios dependent on age at presentation (Table 1) [17].

### 2.2. Diagnostic Challenges in the AYA Population

Medulloblastoma rarity in AYAs contributes to substantial diagnostic delays compared to younger children. Classic cerebellar symptoms, including ataxia and hydrocephalus, occur in only 60% of AYA cases versus 85% in children [18]. Median time to diagnosis extends to 4.2 months in AYAs compared to 2.1 months in pediatric populations, reflecting lower clinical suspicion for posterior fossa tumors in this age group [18]. Delayed recognition frequently results in more advanced disease at presentation, with 35% of AYA patients presenting with metastatic disease compared to 25% in pediatric cohorts [18,19]. Most commonly reported symptoms in AYA medulloblastoma patients include nausea and vomiting (60%), headache (43%), and ataxia (40%) [20,21]. However, symptom presentation may be more subtle and nonspecific compared to younger children, contributing to diagnostic uncertainty. Increased intracranial pressure manifestations, including papilledema and sixth cranial nerve palsy, may develop more insidiously in AYA patients due to greater cranial compliance [21].

### 2.3. Clinical Risk Stratification

Traditional risk stratification classifies patients based on age at diagnosis, extent of surgical resection, and metastatic status [22]. Children older than 3 years with minimal residual tumor (<1.5 cm^2^) and absence of neuraxial metastases (M0 status) are classified as standard-risk, achieving >80% long-term survival with contemporary multimodal therapy [23]. High-risk features include age <3 years at diagnosis, metastatic disease at presentation (M1–M4), large cell/anaplastic histology, and subtotal resection with residual tumor exceeding 1.5 cm^2^ [24]. Application of pediatric risk stratification criteria to AYA patients presents inherent challenges. The age criterion becomes less relevant in adolescent and adult populations, while the extent of resection and metastatic status retain prognostic significance. Recent analyses demonstrate that gross total resection and receipt of adjuvant radiotherapy represent favorable prognostic factors in adult medulloblastoma, while large cell/anaplastic histology associates with diminished survival [25].

## 3. Molecular Subgroups: Characteristics and Age-Specific Distributions

### 3.1. WNT-Activated

WNT-activated tumors constitute approximately 10–15% of all medulloblastomas, characterized by nuclear β-catenin accumulation, activating CTNNB1 mutations, and monosomy 6 [26,27]. These tumors demonstrate the most favorable prognosis among all subgroups, with 5-year overall survival consistently exceeding 90–95% [28,29]. WNT-activated tumors arise predominantly in children and occasionally in adults, but rarely present in infants [30]. The cellular origin of WNT tumors differs fundamentally from other subgroups, arising from lower rhombic lip progenitors or dorsal brainstem precursor cells rather than cerebellar granule neuron progenitors [31]. This distinct developmental origin contributes to characteristic midline localization involving the fourth ventricle, with frequent extension into the cerebellopontine angle and brainstem [32]. In AYA populations, WNT-activated tumors maintain their exceptionally favorable prognosis. Remarkably, TP53 mutations—which confer devastating prognosis in SHH-activated tumors—do not adversely affect outcomes in the WNT subgroup [33,34]. This differential response may reflect the capacity of constitutive β-catenin activation to abrogate radioresistance typically conferred by p53 pathway disruption [35]. Current international clinical trials are investigating reduced-intensity therapeutic approaches for WNT-activated medulloblastoma, recognizing the potential to achieve excellent survival while minimizing long-term neuro-cognitive and endocrine sequelae associated with conventional therapy [36].

### 3.2. SHH-Activated

SHH-activated tumors represent only about 30% of medulloblastomas, and they develop from cerebellar granule neuron precursors due to the constitutive activation of the sonic hedgehog signaling pathway [37,38]. These tumors have a tendency to be laterally localized in the cerebellar hemispheres, which is a notable feature given that other subgroups typically originate in the midline [39]. The age distribution of SHH-activated tumors is bimodal and very distinct, as these tumors are mostly seen in infants (<3 years) and adults (>17 years), while in children aged 3–16 years, they only make up about 10–15% of the cases [40,41]. In the AYA population, SHH-activated tumors are the main molecular subgroup, making up over 60% of the cases [42]. The complete hedgehog signaling pathway is affected through mutations either germline or somatic in the majority of SHH tumors. The typical variations in the hedgehog pathway that are involved in tumor development include PTCH1 mutations resulting in loss of normal function, SMO mutations leading to gain of function, and SUFU mutations causing loss of function [43,44]. In AYA patients, PTCH1 mutations are almost twice as frequent as in pediatric cases, occurring in about 40–50% of SHH tumors versus 20–30% respectively [45,46]. Recent comprehensive methylation profiling has subdivided SHH-activated medulloblastomas into four molecular subtypes with distinct age distributions and clinical characteristics [47]:

SHH-α: Mostly children aged 3–16 years are affected by this subtype, which is the highest-risk SHH subtype, featuring frequent occurrences of TP53 mutations, MYCN and GLI2 amplifications, and chromosomal deletions of 9q, 10q, and 17p. The prognosis of this subtype is worse than that of any other SHH variant [48].

SHH-β: Occurs primarily in infants, exhibits high rates of metastatic disease, and associates with focal PTEN deletions, demonstrating inferior survival compared to SHH-γ [49].

SHH-γ: Presents in infancy and is enriched for medulloblastoma with extensive nodularity (MBEN) histology, representing a favorable-risk group potentially suitable for therapy de-escalation strategies [50].

SHH-δ: Predominantly affects adults, demonstrates enrichment for TERT promoter mutations (occurring in 15–20% of AYA cases), and generally associates with a favorable prognosis [51,52].

### 3.3. Group 3

Group 3 tumors constitute approximately 25 and 27% of all cases of medulloblastoma, and they show the most aggressive biological behavior and the worst clinical outcome [53,54,55]. They also have a strong male predominance (2:1 male/female ratio) and occur almost exclusively in infants and young children; they are exceptionally rare in adolescents and virtually absent in adults [55,56]. A meta-analysis that included 550 patients with medulloblastoma showed that only 6% of adult patients were classified as Group 3 tumors, further demonstrating the extreme rarity of this tumor type once past early childhood [10,57]. The age-restricted distribution of this subtype is suggestive of significant differences in their cellular origins and/or oncogenic requirements [58]. Approximately 40–50% of Group 3 tumors have distant metastases at diagnosis. They typically have large cell/anaplastic histology and demonstrate MYC amplification in approximately 15–20% of cases [10,40,59,60]. The most validated prognostic biomarker within Group 3 tumors is MYC amplification, which is associated with very poor outcomes, with 5-year overall survival ranging from 20 to 45% even with the use of intensive multi-modal therapy [10,61,62]. Intra-tumoral heterogeneity was described in a recent single-cell transcriptomic analysis of Group 3 tumors, identifying distinct cell clusters demonstrating highly aggressive phenotype characteristics and elevated marker expression (e.g., GRM8, AP1S2) correlated with worse prognosis [63]. The MYC oncogene activates a downstream molecular cascade that induces complex metabolic reprogramming, including the upregulation of glycolysis, glutamate metabolism, and the pentose phosphate pathway [64].

### 3.4. Group 4

Group 4 tumors represent the most common molecular subgroup, comprising 35–40% of all cases [65]. These tumors occur across all age ranges but predominate during childhood and adolescence. Like Group 3, Group 4 demonstrates male predominance and frequently presents with metastatic disease [10,66]. Molecular drivers of Group 4 remain incompletely characterized compared to other subgroups. Characteristic genetic features include isochromosome 17q (i17q) in 70–80% of cases, tandem duplications of SNCAIP, and occasional MYCN amplifications [67,68]. Unlike Group 3, MYC amplifications are exceedingly rare in Group 4 [68,69]. Group 4 tumors demonstrate intermediate prognosis, with 5-year overall survival rates ranging from 75 to 90%, superior to Group 3 but inferior to WNT-activated tumors [68,69,70,71]. Recent investigations identified molecular biomarkers capable of stratifying risk within Group 4, including FSTL5 expression patterns distinguishing high-risk from standard-risk patients [47,72].

## 4. Critical Molecular Biomarkers in AYA Medulloblastoma

### 4.1. TP53 Mutations: Subgroup-Specific Prognostic Implications

TP53 mutations occur in approximately 10–16% of all medulloblastomas but demonstrate striking subgroup-specific distributions and prognostic implications [13,73,74]. The prognostic significance varies dramatically according to molecular subgroup, age at diagnosis, and germline versus somatic mutation status (Table 2) [13,75].

In WNT-activated tumors, TP53 mutations occur in approximately 15–20% of cases but do not adversely affect the excellent prognosis characteristic of this subgroup [13,35,76,77]. Multivariate analyses consistently demonstrate that WNT patients with TP53 mutations maintain >90% 5-year survival, comparable to TP53 wild-type WNT cases [78]. This remarkable tolerance may reflect constitutive WNT pathway activation overriding p53-mediated cellular checkpoints [13,79]. In stark contrast, TP53 mutations in SHH-activated tumors confer a devastating prognosis, particularly in pediatric and adolescent age groups [13,80,81]. SHH tumors with TP53 mutations occur predominantly in children aged 5–18 years (median age approximately 9–15 years), contrasting with the bimodal age distribution of TP53 wild-type SHH cases. Approximately 21% of SHH tumors harbor TP53 mutations, with enrichment in the SHH-α molecular subtype [13,82,83,84]. TP53-mutated SHH tumors demonstrate 5-year overall survival of only 41% (±9%), compared to 81% (±5%) survival in TP53 wild-type SHH tumors of similar age groups (*p* < 0.001) [13]. More than 50% of TP53 mutations in SHH tumors represent germline alterations, frequently occurring in the context of Li-Fraumeni syndrome [85,86]. Germline TP53 mutations associate with particularly adverse outcomes, with TP53 mutations accounting for approximately 72% of deaths in children older than 5 years with SHH medulloblastoma [13]. TP53-mutated SHH tumors exhibit distinctive biological characteristics explaining their clinical aggressiveness. These tumors demonstrate the highest overall mutational burden among all medulloblastomas, frequent chromosomal instability including chromothripsis (catastrophic DNA rearrangements), and co-occurrence with MYCN and GLI2 amplifications. TP53 mutations in SHH tumors typically occur in association with downstream pathway alterations rather than upstream PTCH1, SMO, or SUFU mutations, suggesting these tumors are unlikely to respond to smoothened inhibitors currently in development [10,87,88,89,90]. In Group 3 and Group 4 tumors, TP53 mutations are exceptionally rare, occurring in less than 1–2% of cases [13,91,92]. Current risk stratification schemas for SHH-activated tumors incorporate TP53 mutation status as a critical determinant, classifying patients into three risk categories: standard-risk (TP53 wild-type, non-metastatic, MYCN non-amplified), high-risk (metastatic and/or MYCN-amplified), and very high-risk (TP53-mutated, either germline or somatic) [12,93,94].

### 4.2. MYCN and MYC Amplifications

MYCN amplification occurs in approximately 5–10% of all medulloblastomas and demonstrates subgroup-specific distributions and prognostic implications. MYCN amplifications occur primarily in SHH-activated and Group 4 tumors, while MYC amplifications are largely restricted to Group 3 tumors [95,96,97]. In SHH-activated tumors, MYCN amplification occurs in approximately 8–15% of cases and is associated with significantly diminished survival, particularly when accompanied by TP53 mutations [98,99]. MYCN amplification frequently co-occurs with GLI2 amplification in the SHH-α subtype affecting older children and adolescents [100]. While all SHH tumors demonstrate elevated MYCN expression due to hedgehog pathway activation, only tumors with copy number amplification demonstrate adverse prognosis, emphasizing the critical distinction between pathway-driven expression and oncogenic amplification [27,101]. Group 3 tumors have a 15–20% incidence of MYC amplification, making it the strongest negative prognostic factor; associated 5 year survival rates are <50% [51,102,103]. MYC-amplified Group 3 tumors have the highest rate (50%) of metastatic disease and show a propensity for large cell (anaplastic) histology. MYC-amplified Group 3s also have recently been subdivided into high-risk and very high-risk categories based on their MYC status, which is responsible for most treatment failures of the patient within this subgroup of medulloblastomas [12,104,105]. MYC is involved in activated oncogene function by modulating metabolic processes such as increasing glycolysis, changing glutamate metabolism, and activating the pentose phosphate cycle. MYC increases the expression levels of some metabolic enzymes through direct regulation of the same, including transketolase, which has been shown to promote tumor cell growth while inhibiting apoptotic cell death due to oxidative stress [64,106,107]. Recent large-scale multi-variant comparison analyses across multiple medulloblastoma subtypes confirm that TP53 mutations, MYCN amplification, and high-risk clinical features (high-risk clinical features include having disease that is metastatic and subtotal surgical resection) are all independent of one another in both medulloblastoma subtypes recurrence and mortality [12,104,108,109].

### 4.3. Additional Prognostic Molecular Alterations in AYA Patients

TERT promoter mutations represent a molecular alteration of considerable interest in adolescent and young adult medulloblastoma. These mutations affect the gene encoding telomerase reverse transcriptase and are detected in 15–20% of cases within this age group, showing particular enrichment in tumors harboring SHH pathway alterations. From a biological standpoint, these genetic changes enable neoplastic cells to maintain telomere length indefinitely, thereby conferring replicative immortality and circumventing normal senescence mechanisms. Interestingly, several clinical series have documented improved survival outcomes among patients whose tumors harbor TERT promoter mutations, a phenomenon potentially attributable to heightened susceptibility to DNA-damaging therapeutic agents [52,110,111]. Defects in DNA repair mechanisms constitute another clinically relevant molecular feature in this patient population. Homologous recombination deficiency, frequently resulting from alterations in BRCA1/BRCA2 pathway components, occurs in approximately 8–12% of adolescent and young adult cases. This molecular phenotype creates specific therapeutic vulnerabilities, particularly to PARP inhibitor compounds and platinum-containing chemotherapy regimens. Although less common, mismatch repair deficiency is observed in 2–3% of tumors and may serve as a predictive biomarker for response to immune checkpoint blockade, given its association with elevated tumor mutational burden. Chromosomal imbalances demonstrate established prognostic relevance across medulloblastoma molecular subgroups. Monosomy of chromosome 6 correlates with favorable outcomes and shows enrichment in WNT-activated tumors, while isochromosome 17q represents a recurrent finding in Group 4 medulloblastomas [18,27,112,113,114,115]. Conversely, deletions affecting chromosomal arms 9q, 10q, and 17p associate with adverse prognosis, particularly in SHH tumors harboring concurrent TP53 mutations [61,114,115].

### 4.4. Epigenetic and Epitranscriptomic Regulation in AYA Medulloblastoma

The molecular pathogenesis of medulloblastoma extends considerably beyond the canonical framework of somatic DNA mutations, encompassing a rich landscape of epigenetic and post-transcriptional regulatory mechanisms that profoundly influence tumor biology, treatment response, and clinical outcomes. The integration of these multi-layered regulatory processes in a single pathogenic model is becoming more and more recognized as necessary for a complete understanding of the biology of different subgroups, especially AYA patients whose epigenetic landscape reflects age-dependent features.

Non-coding RNAs are a functionally diverse class of regulatory molecules that play crucial roles in the pathogenesis of medulloblastoma. MicroRNAs (miRNAs), short (~22 nucleotide) single-stranded RNAs that suppress target gene expression by either translational repression or mRNA degradation, show subgroup-specific expression patterns.

The miR-17/92 cluster, which is transcriptionally activated by MYC and MYCN, supports cell proliferation and survival in Group 3 and SHH-activated tumors through repression of PTEN and other tumor suppressors, thus converging on the PI3K/AKT/mTOR pathway. On the other hand, miR-124 and miR-128 act as tumor suppressors in medulloblastoma by regulating CDK6, PTBP1, and members of the SHH pathway; their epigenetic silencing through promoter methylation is predominantly found in high-risk SHH and Group 3 subgroups. Long non-coding RNAs (lncRNAs) are yet another functionally significant regulatory modality. By recruiting the PRC2 complex, thereby causing histone H3K27 trimethylation, HOTAIR mediates transcriptional repression of developmental regulatory genes and is associated with aggressive phenotypes of SHH and Group 3 medulloblastomas. H19, a well-known imprinted lncRNA, acts as a competing endogenous RNA (ceRNA) by sequestering miR-675 and therefore regulates IGF2 signaling, which is one of the ways it contributes to the characteristic growth patterns of Group 4 tumors. Additionally, RNA splicing alterations serve as a mechanism of dysregulation, causing functional abnormalities in medulloblastoma. KDM6A is a histone demethylase and Group 4 tumor suppressor, which undergoes abnormal alternative splicing to produce dominant, negative isoforms that hinder the correct H3K27me3 dynamics [115].

Mutations of splicing factors, including alteration of SF3B1 and U2AF1 genes, have been found in a subset of medulloblastoma. In addition, these mutations produce novel peptide sequences via new splicing and can be recognized as neoantigens, thus possibly affecting the immunotherapy responsiveness of these tumor cells. Epitranscriptomic modifications, or chemical modifications of RNA molecules that are distinct from the underlying nucleotide sequence, are being recognized as having significant functional roles in brain tumor biology. N6-methyladenosine (m6A), the most abundant internal modification in eukaryotic mRNA, modulates mRNA stability, translation efficiency, and nuclear export through the coordinated activity of writer (METTL3/METTL14), eraser (FTO/ALKBH5), and reader (YTHDF1/2/3) proteins. In medulloblastoma, elevated METTL3 activity promotes m6A deposition on transcripts encoding hedgehog pathway components, enhancing their translational output through YTHDF1-mediated mechanisms and thereby amplifying SHH signaling in a subgroup-specific manner [116].

In preclinical models, treatment with METTL3 antagonists reduces SHH medulloblastoma cell proliferation, thus revealing m6A machinery as a possible target for therapy. The patterns of histone modifications vary greatly between molecular subgroups and represent a regulatory layer that is additional to the changes in gene expression. The methylation of histone H3K27 trimethylation, a process through which the PRC2 complex suppresses transcription by EZH2, keeps the silencing of the developmental genes and is lost in Group 4 tumors due to the mutation of the KDM6A gene.

**Figure 1:**
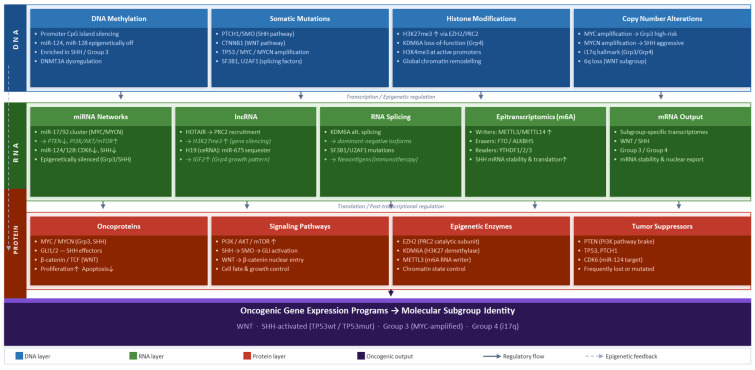
Multi-omics integration schema: DNA–RNA–protein regulatory axis in medulloblastoma. up arrow: increase expression; down arrow: decrease expression.

EZH2 inhibitors (tazemetostat) have been shown to have an effect in laboratory models of medulloblastoma with PRC2 dysregulation, thus justifying the clinical trial of molecularly selected patients. A graphical representation of the deregulation mechanisms that affect each molecular subgroup is summarized in Figure 1. The figure shows how molecular events at the DNA, RNA, and protein levels through a range of epigenetic and post-transcriptional mechanisms, transcriptional mechanisms such as DNA methylation, histone modification, non-coding RNA networks, splicing regulation, and epitranscriptomic marks, all lead to the activation of the main oncogenic gene expression programs of each molecular subgroup [115,116].

## 5. Treatment Approaches

### 5.1. Surgical Management

The main treatment for medulloblastoma for all ages is the maximum safe surgical resection. The surgery objectives include obtaining a histological diagnosis, molecular characteristics, cytoreduction, and restoring the flow of cerebrospinal fluid [109,116,117]. The extent of resection greatly affects prognosis. Gross total resection (defined as having no residual disease in postoperative imaging) or near-total resection (defined as having residual tumor volume of less than 1.5 cm^2^) has resulted in better prognoses than subtotal resection results [109,118]. In adults, gross total resection (as an independent prognostic factor) has been shown to have significantly better overall survival rates at five years than those with subtotal resection [119]. However, any attempt to achieve aggressive resections must weigh the potential benefits against the risk of posterior fossa syndrome (cerebellar mutism), cranial nerve deficits, and injury to the brainstem, particularly for tumors involving critical structures [120]. Modern neurosurgical techniques include intraoperative neurophysiological monitoring (such as brainstem auditory evoked potentials and electromyography) to reduce the likelihood of neurologic morbidity due to the surgical resection of the tumor. In select cases where there is hydrocephalus due to obstruction, an endoscopic third ventriculostomy may be performed, thus avoiding the need for permanent shunting of the ventricles [121,122].

### 5.2. Radiation Therapy

Craniospinal irradiation (CSI) with posterior fossa or tumor bed boost represents a critical component of curative therapy for medulloblastoma in patients older than 3 years [23,123]. Standard-risk patients usually receive 23.4–24 Gy CSI and 54–55.8 Gy boost to the posterior fossa, while high-risk patients have higher doses of 36–39.6 Gy CSI as part of their treatment [124,125]. AYA patients respond to radiotherapy differently than younger pediatric patients because their ability to tolerate radiation and develop long-term neuro-cognitive effects is different. Contemporary techniques such as intensity-modulated radiation therapy (IMRT) and proton therapy produce excellent local control in AYA patients and may reduce potential long-term neuro-cognitive and endocrine complications in these patients [126,127]. There is consistent evidence that receiving adjunctive radiation is important for adults with medulloblastoma, which leads to improved prognosis; this is evidenced by the negative effect of omitting radiation therapy on survival [128,129]. The theoretical dosimetric advantages of proton therapy compared to traditional photon-based radiation therapy may lower the total dose delivered to developing brain tissue and may lower the risk of neuro-cognitive deficits, endocrine dysfunction, and secondary cancers [130].

### 5.3. Chemotherapy

The usage of adjuvant chemotherapy in adult standard-risk medulloblastoma cases has been a debate among specialists for years; a growing body of evidence now indicates that chemotherapy should be included systemically in adults with medulloblastoma even if they have non-metastatic disease [25,131]. Propensity score analyses in national cancer databases have demonstrated that radiation plus upfront chemotherapy (CT) yields a significant increase in OS rates at 5 years as compared with just receiving radiation alone (84% vs. 74%, *p* = 0.01), with particular benefit observed in M0 patients receiving full-dose CSI [132]. AYA medulloblastoma patients receive standard chemotherapy regimens adapted primarily from using pediatric protocols; typical regimens employ large dose platinum-based combination drugs including Cisplatin, Vincristine, CCNU (lomustine) and Cyclophosphamide. The most common regimens are based upon the “Packer” regimen (Vincristine and Cisplatin given during RT followed by maintenance therapy of Cyclophosphamide, Cisplatin and Vincristine) [23,133,134]. For the management of high-risk and recurrent medulloblastoma patients, the use of high-dose chemotherapy in conjunction with the reinfusion of autologous stem cells has been considered. Several studies have shown that this approach is a safe and effective treatment option for select high-risk and relapsed patients with medulloblastoma; the myeloablative regimens employed (usually containing Thiotepa, Carboplatin and Etoposide) enhance the use of HSCT by potentially overcoming mechanisms of chemorefractory tumor cells. As a population, adult medulloblastoma patients may also experience a greater frequency of treatment-related toxicity and discontinuation of treatment early than do pediatric medulloblastoma patients, requiring the use of individualized dose adjustments for all adult patients [135,136,137]. Despite these issues, the survival outcomes for adult and pediatric-inspired/adapted regimens of therapy are comparable to each other and suggest that it is possible to implement intensive multimodal therapies in AYA patients with medulloblastoma [138].

**Figure 2:**
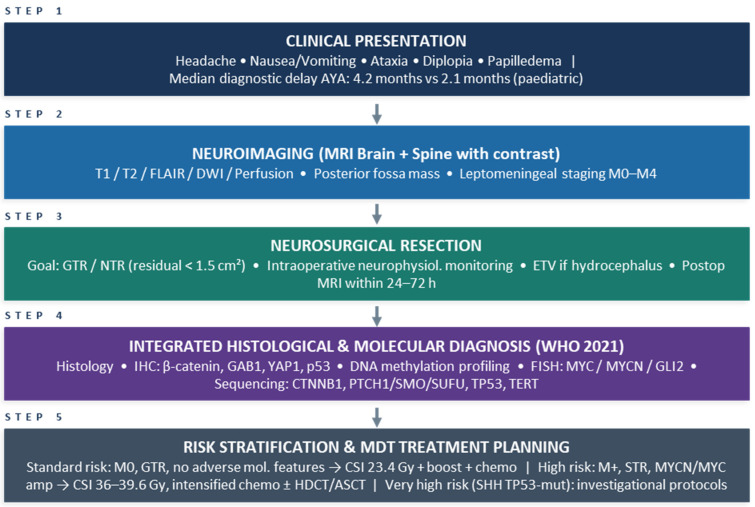
Diagnostic algorithm.

In Figure 2, a diagnostic algorithm for medulloblastoma in AYAs is proposed. Following initial MRI and neurosurgical resection, integrated histological and molecular analysis—including DNA methylation profiling, immunohistochemistry, and targeted sequencing—allows classification into the four WHO 2021 molecular subgroups. Key AYA-specific biomarkers (TP53, MYCN, TERT promoter) are highlighted at each relevant decision node to guide risk stratification and treatment planning [9].

Abbreviations: GTR, gross total resection; NTR, near-total resection; STR, subtotal resection; ETV, endoscopic third ventriculostomy; IHC, immunohistochemistry; FISH, fluorescence in situ hybridization; LCA, large cell/anaplastic; HDCT, high-dose chemotherapy; ASCT, autologous stem cell transplantation; CSI, craniospinal irradiation; MDT, multidisciplinary team.

### 5.4. Molecular Subgroup-Directed Therapies

The dominance of SHH-activated medulloblastomas among AYAs, particularly those caused by PTCH1 or SMO mutations, opens up the possibility of treating the hedgehog signaling pathway therapeutically. Smoothened antagonists, mainly vismodegib and sonidegib, have demonstrated effective anticancer activity in preclinical experimental systems and early human trials [17,139,140,141]. Clinical response to SMO inhibitors seems to differ according to patients’ age. Adult patients whose tumors carry mutations of upstream pathway components usually get more benefit from these treatments than children with mutations of downstream genes like SUFU or GLI1. Besides that, TP53-mutated SHH medulloblastomas represent a major therapeutic dilemma. Besides the fact that they usually have MYCN and GLI2 amplification, two genes located downstream of smoothened antagonists in the signaling pathway, they therefore lack sensitivity to the available hedgehog pathway antagonists [42,89,141,142,143]. Latest translational investigations have pinpointed several epigenetic regulatory mechanisms that may serve as therapeutic targets, which could work in MYC-driven Group 3 tumors as well [106,144]. Besides the fact that they have demonstrated anti-cancer effects in models with MYC amplification, BET protein inhibitors, compounds including CI-994 that target histone deacetylases, and agents that inhibit SETD8 methyltransferase activity, have all been investigated in the same context [97,145,146]. These results imply that the therapeutic modulation of chromatin regulatory mechanisms could potentially become a treatment for certain molecular medulloblastoma subsets, although clinical validation remains necessary.

A critical limitation of smoothened inhibitors in clinical practice is the emergence of both primary and acquired resistance, severely constraining their therapeutic utility. Primary resistance is most frequently observed in tumors harboring downstream pathway alterations: amplification of GLI2 or MYCN, loss-of-function mutations in SUFU, and chromosomal gains at 2q—all of which sustain transcriptional output of the hedgehog pathway independent of SMO activity. These molecular features, which characterize the SHH-α subtype and are enriched in adolescent patients, explain the limited clinical benefit of vismodegib and sonidegib in this specific subset despite pathway dependence at the level of SMO [139,140,141]. Acquired resistance following initial SMO inhibitor response has been attributed to multiple mechanisms. Point mutations within the SMO ligand-binding pocket (most notably the D473H substitution) directly impair drug binding. Additionally, pathway reactivation through non-canonical mechanisms—including activation of the PI3K/AKT/mTOR axis, WNT pathway co-activation, and epigenetic reprogramming of GLI target gene accessibility—has been documented in experimental models of acquired vismodegib resistance [141,142]. In MYC-amplified Group 3 tumors, intrinsic resistance to conventional targeted agents is compounded by metabolic reprogramming, notably enhanced glycolysis and oxidative phosphorylation, which sustain tumor cell survival under therapeutic stress. Epigenetic plasticity, including dynamic alterations in histone acetylation mediated by BRD4, further enables transcriptional adaptation and phenotypic switching. Rational combination strategies currently under investigation aim to address these resistance mechanisms by cotargeting SMO with GLI inhibitors (GANT61), PI3K/mTOR inhibitors, and Aurora kinase inhibitors, and by combining BET bromodomain inhibitors with CDK4/6 inhibitors in MYC-dependent subtypes [141,142,143,144,145,146].

## 6. Survival Outcomes in AYA Medulloblastoma

Systematic reviews assessing outcomes in AYA patients reveal considerable variations in reported survival rates that mirror differences in patient cohort, treatment modalities, and molecular profiling [147]. Five-year overall survival rates vary between 40 and 89%, whereas disease-free survival ranges between 49 and 89%. Population-based studies offer a great perspective of the “real-world” outcomes. The first SEER database study of 454 adult patients diagnosed between 1973 and 2004 showed 5-year and 10-year overall survival rates of 64.9% and 52.1%, respectively. A more recent SEER analysis that included 349 patients diagnosed until 2013 showed an estimated 5-year and 10-year survival rates of 74.2%, and 67.3%, respectively, which were significantly influenced by the extent of resection, receipt of radiotherapy and chemotherapy, and histological subtype [15,148,149]. Subgroup-specific survival outcomes demonstrate marked heterogeneity. WNT-activated tumors maintain excellent prognosis across age groups, with 5-year survival exceeding 90–95% [28]. SHH-activated tumors demonstrate variable outcomes dependent on TP53 mutation status, with TP53 wild-type cases achieving 70–80% 5-year survival while TP53-mutated cases experience only 40–50% survival [13]. Group 3 tumors demonstrate the worst outcomes, with 5-year survival below 50%, particularly in MYC-amplified cases [13,102]. Age at diagnosis influences survival outcomes within molecular subgroups. Analysis of 550 medulloblastoma patients stratified by age demonstrated that adult SHH patients achieved 5-year overall survival of 81%, significantly superior to pediatric SHH cohorts with TP53 mutations [41]. This survival advantage reflects the lower prevalence of TP53 mutations in adult SHH cases and enrichment for the favorable-prognosis SHH-δ subtype [47]. Metastatic status at diagnosis represents a critical prognostic determinant across all age groups. Approximately 25–35% of new diagnoses in the AYA group are metastatic at presentation, which is similar to or slightly higher than the pediatric group [3,4,14]. With risk-adapted therapeutic approaches and molecular profiling, treatment results in the AYA population have improved dramatically in recent years. Some recent single-institution series have demonstrated that 5-year overall survival reaches up to 70–75% for unselected AYA medulloblastoma patients, whereas progression-free survival is 60–65% [129,136,150,151].

### Patterns of Failure and Salvage Strategies

Recurrent medulloblastoma presents formidable therapeutic challenges, with median survival following recurrence ranging from 6 to 12 months across published series [152]. Failure patterns depict subgroup-specific molecular profiles as Group 3 tumors with high early systemic and leptomeningeal progression rates, whereas SHH tumors more often show delayed local recurrence [58,153]. There is insufficient consensus on salvage therapy to treat the recurrent medulloblastoma, and these include re-irradiation (for patients with a sufficient time interval since first radiotherapy), high-dose chemotherapy with stem cell rescue, targeted molecular therapies, and experimental immunotherapeutic approaches [154,155,156]. Local recurrences treated with re-irradiation using stereotactic radiosurgery or conformal techniques have shown to be feasible in select cases, and local control rates of 30–50% were obtained with acceptable toxicity profiles. High-dose chemotherapy followed by autologous stem cell rescue is a well-recognized salvage procedure, especially for those who did not undergo the treatment modality at frontline therapy [135,155,157].

## 7. Survivorship Challenges and Long-Term Sequelae

Survivors of AYA medulloblastoma endure significant long-term health problems due to chronic medical conditions, as well as changes in neuropsychological function and social functioning, which have a strong negative effect on their quality of life. Due to the unique developmental status of AYAs, including transitioning through school, beginning careers, establishing intimate relationships, and planning for family, the effects of treatment-related sequelae are amplified [158,159,160].

### 7.1. Neuro-Cognitive Effects

The neuro-cognitive deterioration following craniospinal irradiation is dose-dependent and impairs the functioning of working memory, processing speed, and executive function domains [160]. Younger patients at the time of treatment tend to exhibit more severe neuro-cognitive impairments. Still, even AYA patients show significant neuro-cognitive decline when compared with age-matched controls. In addition, neuro-cognitive follow-up of medulloblastoma survivors points to the patient’s condition worsening in several areas over time, with children being especially vulnerable during phases of rapid developmental change. AYA survivors had lower levels of qualifications, were less frequently employed, and depended more on their families compared to sibling controls [161,162,163].

### 7.2. Endocrine Complications

Post craniospinal irradiation, the hypothalamic–pituitary axis dysfunction is the most common endocrine sequelae, seen in almost half of the survivors (40–50%). Growth hormone deficiency is the major abnormality, followed by thyroid dysfunction, adrenal insufficiency, and hypogonadotropic hypogonadism. Adult survivors are at a higher risk of metabolic syndrome, osteoporosis, and cardiovascular disease, which are directly or indirectly related to their endocrine dysfunction [164,165,166].

### 7.3. Ototoxicity and Auditory Complications

Ototoxicity is an outcome of the cumulative effects of cisplatin-based chemotherapy and posterior fossa radiation therapy, which produces high-frequency sensorineural hearing loss in 30 to 60% of survivors. Hearing impairment affects AYAs’ quality of life, educational achievements, and social functioning, particularly when they are attempting to transition into the workforce or continue their education [167,168].

### 7.4. Secondary Malignancies

Long-term survivors also have a significantly increased risk of secondary malignancies, with the cumulative incidence of secondary malignancies approaching 2 to 4% in the 20 years post-diagnosis [169]. Secondary malignancies induced by radiation therapy are most commonly meningiomas, gliomas, and various sarcomas that develop within or at the border of the radiated field. Patients with germline mutations of TP53 (Li-Fraumeni syndrome) have an especially increased risk of developing secondary malignancy and should be placed on a higher-intensity surveillance protocol [170,171].

### 7.5. Psychosocial and Quality of Life Considerations

AYA cancer survivors have an increased risk of depression, anxiety and post-traumatic stress disorder than age-matched controls. Survivors of medulloblastoma have reported decreased health-related quality of life in a variety of domains, including physical functioning, social relationships, and emotional well-being [172,173,174]. Fertility preservation is an important concern for AYAs, as alkylating chemotherapy agents and craniospinal radiation can impact their reproductive abilities. Proactive fertility counseling and cryopreservation options should be included in the treatment planning of all AYAs [174,175].

## 8. Future Directions and Research Priorities

### 8.1. Enhanced Molecular Characterization

By using comprehensive integrative genomic profiling, which combines many technologies, including whole-genome sequencing with transcriptomics (RNA sequencing), DNA methylation analyses, and single-cell technologies, the classification of medulloblastomas will continue to become more sophisticated and define new therapeutic targets. Many studies have shown that there is a significant amount of intratumoral heterogeneity in the recurrence of medulloblastomas, as well as a large amount of change in molecular characteristics at the time of recurrence. Thus, repeated molecular profiling is critical in relapsed diseases [63,92,176,177]. Liquid biopsy methods assessing circulating tumor DNA from both cerebrospinal fluid and blood samples represent minimally invasive means to monitor disease, detect molecular recurrence at an early stage, and assess resistance mechanisms [178,179,180]. Implementation of liquid biopsy technologies in clinical practice could allow adaptive treatment strategies and personalized treatment modifications.

### 8.2. Novel Therapeutic Strategies

In the past, medulloblastoma has been viewed as an immunologically quiescent tumor. However, new evidence is surfacing that shows that medulloblastomas have tumor-infiltrating lymphocytes and therefore may have opportunities for immune treatment [181]. Immunotherapy with PD-1 or CTLA-4 antibodies is now in clinical trials in patients with recurrent medulloblastoma and is being primarily studied in patients with tumors that have high mutation rates. Chimeric antigen receptor (CAR) T-cell therapy that targets glypican-2 and B7-H3 expressed on the surface of medulloblastomas is also undergoing investigative studies in early-phase clinical trials [182,183]. Research into new drug therapies for medulloblastoma is progressing rapidly and significantly expanding on the current therapeutic strategy of using hedgehog pathway antagonists to treat this cancer type. There is now a large number of targeted agents in various stages of preclinical and clinical testing against molecular targets that are meaningful to medulloblastoma. This includes: cyclin-dependent kinase (CDK) 4/6 inhibitors for tumors with aberrant cell cycling, PARP inhibitors for tumors with a defective homologous recombination (HR) pathway, and Aurora kinase inhibitors for MYC-amplified tumors [184,185]. All of these different agents exploit unique molecular dependencies that define groups of medulloblastoma patients.

Group 3 medulloblastomas display unique metabolic changes that could lead to potential targeting in future treatment. Specifically, these tumors have metabolic dependency on glycolysis, glutamate metabolism and oxidative phosphorylation via mitochondria as their means to produce ATP [64,186]. Experimental studies using compounds that inhibit transketolase or glutaminase have yielded encouraging results in laboratory models driven by MYC oncogene amplification [187,188]. Such metabolic targeting strategies represent a conceptually distinct therapeutic approach that addresses fundamental bioenergetic requirements of aggressive medulloblastoma variants.

### 8.3. Clinical Trial Participation

AYAs diagnosed with medulloblastoma are significantly underrepresented in clinical trial research, such that less than 10% of qualified AYAs have been included in studies for the treatment of medulloblastoma [189]. The lack of participation can be attributed to several barriers. Each participating in medulloblastoma therapeutic studies has included the limited availability of trials appropriate for the age of the patient, restrictive eligibility criteria, and a lack of collaboration between pediatric and adult oncologists providing care. Collaborative international groups such as the International Medulloblastoma Working Group and specific AYAs will develop clinical trials directed toward the AYA population as part of their collaborative relationships [190,191]. Modern clinical trials are designed using risk-adapted therapies incorporating molecular markers; some patients are treated with reduced intensity therapies, and some patients with high-risk disease are treated with intensified or targeted therapies [192].

Future clinical trials enrolling AYA patients with medulloblastoma should incorporate several design elements specifically addressing the biological and clinical characteristics of this population. First, molecular eligibility criteria must be defined prospectively, with mandatory pre-treatment classification by the WHO 2021 integrated diagnosis, including subgroup assignment and assessment of TP53 mutation status, MYCN/MYC amplification, and TERT promoter mutation [141]. Stratified randomization by molecular subgroup and metastatic status is essential to ensure meaningful subgroup analyses, given the relative rarity of non-SHH subtypes in this age group. Second, liquid biopsy should be incorporated as a protocol-mandated translational endpoint. Circulating tumor DNA (ctDNA) quantified from plasma and cell-free DNA from cerebrospinal fluid offers minimally invasive opportunities for real-time disease monitoring, assessment of molecular response during treatment, and early identification of resistance-associated alterations. Serial ctDNA sampling at baseline, during treatment, at response assessment, and at relapse would enable characterization of clonal dynamics and identification of emerging resistance mechanisms in a manner that tissue biopsy cannot feasibly provide. Third, patient-reported outcome instruments validated for AYA populations should be integrated as co-primary endpoints alongside traditional radiological and survival endpoints, capturing neuro-cognitive function, fatigue, fertility concerns, and occupational and social functioning—domains particularly relevant during the developmental transitions characteristic of this age group. Fourth, adaptive master protocol designs, such as umbrella or basket trials with pre-specified molecular biomarker-driven interim analyses, represent the most resource-efficient framework for investigating multiple targeted agents across molecular subgroups within a rare tumor population. Finally, mandatory cross-registration between pediatric cooperative groups (e.g., Children’s Oncology Group) and adult cooperative groups (e.g., EORTC) is essential to achieve adequate sample sizes and to ensure consistent treatment delivery and data quality across participating institutions [190,191,192].

### 8.4. Precision Medicine Implementation

The application of extensive molecular profiling within routine clinical practice necessitates establishing a strong diagnostic capacity (including reference labs with medulloblastoma classification expertise and quality-controlled analysis workflows) [193]. Multidisciplinary tumor boards that include molecular pathologists, neuro-oncologists, radiation oncologists, and neurosurgeons allow optimal interpretation of molecular test data and therefore help with treatment planning [194]. The establishment of molecular diagnostic consensus criteria and standard reporting templates creates consistency in classification, which in turn aids in the implementation of these assessments across a range of different health service settings [195]. The 2021 WHO classification of central nervous system tumors has continued to include molecular diagnostic requirements as part of its official classification criteria and clarifies the definitions of groups of tumors based on their integrated multi-omic assessments [9].

## 9. Conclusions

Medulloblastoma in adolescents and young adults essentially shows a different clinical profile that is marked by distinctive molecular features, issues relating to therapy, and consideration of the survivors’ needs, all of which isolate this group from both younger pediatric and older adult patients. The dominance of SHH-activated tumors, age-specific patterns of molecular changes that have a prognostic value, such as TP53 mutations and MYCN amplifications, as well as the varying ability to tolerate treatments, thus, highlight the necessity for age, adjusted therapeutic approaches. A very modern risk stratification that takes into account molecular subgroup classification, TP53 mutation status, MYC/MYCN amplification, and clinical features facilitates a tailored treatment plan and prognostic counseling. The combination of targeted molecular therapies, improved radiation techniques, and risk-adapted chemotherapy regimens potentially allows the survival rate to be elevated, whilst the long-term sequelae that negatively impact the quality of life of people going through a crucial period of development are being kept at a minimum. There are still considerable uncertainties as to the perfect therapeutics for high-risk molecular subgroups, as well as the ways of managing recurrent disease and the long-term survivorship in AYA populations. Increased enrolment in clinical trials, complete molecular profiling, creation of novel targeted therapies, and coordination of multidisciplinary care delivery are the four major essential priorities for advancing outcomes in this underserved patient population. The change from histology-based to integrated molecular classification of medulloblastoma is a prime example of how precision oncology can be truly revolutionary. Further uncovering of molecular pathways responsible for subgroup-specific biology, discovery of drug targets, and application of basic research to clinical practice can help us attain the final objective of curing medulloblastoma with the best possible quality of life for patients at any age, even young adults.

## Figures and Tables

**Table 1:** Molecular subgroups of medulloblastoma: key characteristics and age distributions.

Subgroup	Frequency (%)	Peak Age	Key Genetic Alterations	Prognosis (5-Year OS)	AYA Prevalence
WNT-activated	10–15	8–12 years	CTNNB1 mutations, monosomy 6	>90–95%	10–15%
SHH-activated	~30	Bimodal: <3 years, >17 years	PTCH1, SMO, SUFU mutations; TP53 mutations (21%)	70–80% (TP53 WT); 40–50% (TP53 mut)	60–70%
SHH-α	5–7	3–16 years	TP53, MYCN, GLI2 amplifications	30–50%	Rare
SHH-δ	8–10	Adults	TERT promoter mutations	75–85%	40–50%
Group 3	25–27	<10 years	MYC amplification (15–20%), GFI1/GFI1B alterations	50–60% overall; 20–45% (MYC amp)	<5%
Group 4	35–40	5–15 years	Isochromosome 17q (70–80%), SNCAIP duplications	75–90%	20–25%

Abbreviations: OS, overall survival; WT, wild-type; mut, mutated; amp, amplified; AYA, adolescent and young adult.

**Table 2:** Critical prognostic biomarkers in AYA medulloblastoma.

Biomarker	Affected Subgroup(s)	Frequency in AYA	Prognostic Impact	Therapeutic Implications
TP53 mutation	SHH (primarily), rare in others	8–12% overall; 21% of SHH	Very unfavorable in SHH (HR 2.0–2.5); neutral in WNT	Intensified therapy; avoid radiation in germline cases; investigational agents
MYC amplification	Group 3	Rare (<2% of AYA)	Very unfavorable (HR 2.5–3.0)	BET inhibitors, epigenetic modulators, metabolic targeting
MYCN amplification	SHH, Group 4	5–8% of AYA	Unfavorable (HR 1.5–2.0)	Aurora kinase inhibitors, CDK inhibitors
TERT promoter mutation	SHH-δ predominantly	15–20% of AYA	Variable; potentially favorable in some contexts	May predict radiotherapy sensitivity
Metastatic disease (M+)	All subgroups	25–35% of AYA	Unfavorable (HR 1.8–2.2)	Intensified CSI (36–39.6 Gy), systemic therapy
Gross total resection	All subgroups	Achieved in 60–70%	Favorable (HR 0.5–0.7 for GTR vs. STR)	Maximal safe resection priority
Chromosome 6 monosomy	WNT	80–85% of WNT cases	Favorable (subgroup marker)	De-escalation strategies under investigation
Isochromosome 17q	Group 4 predominantly	70–80% of Group 4	Intermediate; subgroup marker	Risk stratification within Group 4

Abbreviations: HR, hazard ratio; CSI, craniospinal irradiation; GTR, gross total resection; STR, subtotal resection.

## Data Availability

No new data were created or analyzed in this study. This review article synthesizes publicly available published literature. All references are cited appropriately to facilitate access to original sources.

## Abbreviations

The following abbreviations are used in this manuscript:

AYA	Adolescent and Young Adult
CNS	Central Nervous System
WNT	Wingless-type (signaling pathway)
SHH	Sonic Hedgehog
WHO	World Health Organization
WT	Wild-Type
SEER	Surveillance, Epidemiology, and End Results
CSI	Craniospinal Irradiation
MBEN	Medulloblastoma with Extensive Nodularity
CDK	Cyclin-Dependent Kinase
PARP	Poly (ADP-ribose) Polymerase
SMO	Smoothened
IMRT	Intensity-Modulated Radiation Therapy
CT	Chemotherapy
CCNU	Lomustine
HSCT	Hematopoietic Stem Cell Transplantation
BET	Bromodomain and Extra-Terminal domain
CAR	Chimeric Antigen Receptor
DNA	Deoxyribonucleic Acid
RNA	Ribonucleic Acid
ATP	Adenosine Triphosphate

## References

[1] Ostrom Q.T., Gittleman H., Liao P., Rouse C., Chen Y., Dowling J., Wolinsky Y., Kruchko C., Barnholtz-Sloan J. (2014). CBTRUS Statistical Report: Primary Brain and Central Nervous System Tumors Diagnosed in the United States in 2007–2011. Neuro Oncol..

[2] Pollack I.F., Jakacki R.I. (2011). Childhood brain tumors: Epidemiology, current management and future directions. Nat. Rev. Neurol..

[3] Sorajja N., Moore K.J., Sample J.M., Hubbard A.K., Williams L.A. (2022). Global variation in young adult central nervous system tumor incidence by region, age, and sex from 1988 to 2012. Cancer Epidemiol..

[4] Smoll N.R., Drummond K.J. (2012). The incidence of medulloblastomas and primitive neurectodermal tumours in adults and children. J. Clin. Neurosci..

[5] Barr R.D., Ferrari A., Ries L., Whelan J., Bleyer W. (2016). Cancer in Adolescents and Young Adults: A Narrative Review of the Current Status and a View of the Future. JAMA Pediatr..

[6] Lim-Fat M.J., Bennett J., Ostrom Q., Touat M., Franceschi E., Schulte J., Bindra R.S., Fangusaro J., Dhall G., Nicholson J. (2025). Central nervous system tumors in adolescents and young adults: A Society for Neuro-Oncology Consensus Review on diagnosis, management, and future directions. Neuro Oncol..

[7] Northcott P.A., Korshunov A., Witt H., Hielscher T., Eberhart C.G., Mack S., Bouffet E., Clifford S.C., Hawkins C.E., French P. (2011). Medulloblastoma comprises four distinct molecular variants. J. Clin. Oncol..

[8] Taylor M.D., Northcott P.A., Korshunov A., Remke M., Cho Y.J., Clifford S.C., Eberhart C.G., Parsons D.W., Rutkowski S., Gajjar A. (2012). Molecular subgroups of medulloblastoma: The current consensus. Acta Neuropathol..

[9] Louis D.N., Perry A., Wesseling P., Brat D.J., Cree I.A., Figarella-Branger D., Hawkins C., Ng H.K., Pfister S.M., Reifenberger G. (2021). The 2021 WHO Classification of Tumors of the Central Nervous System: A summary. Neuro Oncol..

[10] Kool M., Korshunov A., Remke M., Jones D.T.W., Schlanstein M., Northcott P.A., Cho Y.J., Koster J., Schouten-van Meeteren A., van Vuurden D. (2012). Molecular subgroups of medulloblastoma: An international meta-analysis of transcriptome, genetic aberrations, and clinical data of WNT, SHH, Group 3, and Group 4 medulloblastomas. Acta Neuropathol..

[11] Northcott P.A., Buchhalter I., Morrissy A.S., Hovestadt V., Weischenfeldt J., Ehrenberger T., Gröbner S., Segura-Wang M., Zichner T., Rudneva V.A. (2017). The whole-genome landscape of medulloblastoma subtypes. Nature.

[12] Ramaswamy V., Remke M., Bouffet E., Bailey S., Clifford S.C., Doz F., Kool M., Dufour C., Vassal G., Milde T. (2016). Risk stratification of childhood medulloblastoma in the molecular era: The current consensus. Acta Neuropathol..

[13] Zhukova N., Ramaswamy V., Remke M., Pfaff E., Shih D.J.H., Martin D.C., Castelo-Branco P., Baskin B., Ray P.N., Bouffet E. (2013). Subgroup-specific prognostic implications of TP53 mutation in medulloblastoma. J. Clin. Oncol..

[14] Kristeva M., Suprun A., Ghaffar E., Wallis C. (2018). Adult Medulloblastoma: Occurrence of a Rare Event. Cureus.

[15] Ma A.K., Freedman I., Lee J.H., Miyagishima D., Ahmed O., Yeung J. (2020). Tumor Location and Treatment Modality are Associated with Overall Survival in Adult Medulloblastoma. Cureus.

[16] Ostrom Q.T., de Blank P.M., Kruchko C., Petersen C.M., Liao P., Finlay J.L., Stearns D.S., Wolff J.E., Wolinsky Y., Letterio J.J. (2015). Alex’s Lemonade Stand Foundation Infant and Childhood Primary Brain and Central Nervous System Tumors Diagnosed in the United States in 2007–2011. Neuro Oncol..

[17] Cambruzzi E. (2018). Medulloblastoma, WNT-activated/SHH-activated: Clinical impact of molecular analysis and histogenetic evaluation. Childs Nerv. Syst..

[18] Ruggiero A., Attinà G., Talloa D., Mastrangelo S., Romano A., Maurizi P., Chiesa S., Tamburrini G., Olivi A., Albanese A. (2025). Medulloblastoma in Adolescents and Young Adults (AYA): Bridging Pediatric Paradigms and Adult Oncology Practice. J. Clin. Med..

[19] Gajjar A., Bowers D.C., Karajannis M.A., Karajannis M.A., Leary S., Witt H., Gottardo N.G. (2015). Pediatric Brain Tumors: Innovative Genomic Information Is Transforming the Diagnostic and Clinical Landscape. J. Clin. Oncol..

[20] Pui C.H., Gajjar A.J., Kane J.R., Pappo A.S. (2011). Challenging issues in pediatric oncology. Nat. Rev. Clin. Oncol..

[21] Halperin E.C., Friedman H.S. (1996). Is there a correlation between duration of presenting symptoms and stage of medulloblastoma at the time of diagnosis?. Cancer.

[22] Chang C.H., Housepian E.M., Herbert C. (1969). An operative staging system and a megavoltage radiotherapeutic technic for cerebellar medulloblastomas. Radiology.

[23] Packer R.J., Gajjar A., Vezina G., Rorke-Adams L., Burger P.C., Robertson P.L., Bayer L., LaFond D., Donahue B.R., Marymont M.H. (2006). Phase III study of craniospinal radiation therapy followed by adjuvant chemotherapy for newly diagnosed average-risk medulloblastoma. J. Clin. Oncol..

[24] Crawford J.R., MacDonald T.J., Packer R.J. (2007). Medulloblastoma in childhood: New biological advances. Lancet Neurol..

[25] Brandes A.A., Franceschi E., Tosoni A., Blatt V., Ermani M. (2009). Adult neuroectodermal tumors of posterior fossa (medulloblastoma) and of supratentorial sites (stPNET). Crit. Rev. Oncol. Hematol..

[26] Ellison D.W., Onilude O.E., Lindsey J.C., Lusher M.E., Weston C.L., Taylor R.E., Pearson A.D., Clifford S.C. (2005). beta-Catenin status predicts a favorable outcome in childhood medulloblastoma: The United Kingdom Children’s Cancer Study Group Brain Tumour Committee. J. Clin. Oncol..

[27] Clifford S.C., Lusher M.E., Lindsey J.C., Langdon J.A., Gilbertson R.J., Straughton D., Ellison D.W. (2006). Wnt/Wingless pathway activation and chromosome 6 loss characterize a distinct molecular sub-group of medulloblastomas associated with a favorable prognosis. Cell Cycle.

[28] Ellison D.W., Kocak M., Dalton J., Megahed H., Lusher M.E., Ryan S.L., Zhao W., Nicholson S.L., Taylor R.E., Clifford S.C. (2011). Definition of disease-risk stratification groups in childhood medulloblastoma using combined clinical, pathologic, and molecular variables. J. Clin. Oncol..

[29] Shih D.J., Northcott P.A., Remke M., Remke M., Korshunov A., Ramaswamy V., Kool M., Luu B., Yao Y., Wang X. (2014). Cytogenetic prognostication within medulloblastoma subgroups. J. Clin. Oncol..

[30] Gibson P., Tong Y., Robinson G., Thompson M.C., Currle D.S., Eden C., Kranenburg T.A., Hogg T., Poppleton H., Martin J. (2010). Subtypes of medulloblastoma have distinct developmental origins. Nature.

[31] Vladoiu M.C., El-Hamamy I., Donovan L.K., Farooq H., Holgado B.L., Sundaravadanam Y., Ramaswamy V., Hendrikse L.D., Kumar S., Mack S.C. (2019). Childhood cerebellar tumours mirror conserved fetal transcriptional programs. Nature.

[32] Millard N.E., De Braganca K.C. (2016). Medulloblastoma. J. Child. Neurol..

[33] Gajjar A., Robinson G.W. (2014). Medulloblastoma-translating discoveries from the bench to the bedside. Nat. Rev. Clin. Oncol..

[34] Pfaff E., Remke M., Sturm D., Benner A., Witt H., Milde T., von Bueren A.O., Wittmann A., Schöttler A., Jorch N. (2010). TP53 mutation is frequently associated with CTNNB1 mutation or MYCN amplification and is compatible with long-term survival in medulloblastoma. J. Clin. Oncol..

[35] Phoenix T.N., Patmore D.M., Boop S., Boulos N., Jacus M.O., Patel Y.T., Roussel M.F., Finkelstein D., Goumnerova L., Perreault S. (2016). Medulloblastoma genotype dictates blood brain barrier phenotype. Cancer Cell.

[36] Mynarek M., von Hoff K., Pietsch T., Ottensmeier H., Warmuth-Metz M., Bison B., Pfister S., Korshunov A., Schüz J., Lannering B. (2020). Nonmetastatic medulloblastoma of early childhood: Results from the prospective clinical trial HIT-2000 and an extended validation cohort. J. Clin. Oncol..

[37] Wechsler-Reya R.J., Scott M.P. (1999). Control of neuronal precursor proliferation in the cerebellum by Sonic Hedgehog. Neuron.

[38] Yang Z.J., Ellis T., Markant S.L., Read T.A., Kessler J.D., Bourboulas M., Schüller U., Machold R., Fishell G., Rowitch D.H. (2008). Medulloblastoma can be initiated by deletion of Patched in lineage-restricted progenitors or stem cells. Cancer Cell.

[39] Gottardo N.G., Hansford J.R., McGlade J.P., McGlade J.P., Alvaro F., Ashley D.M., Bailey S., Baker D.L., Bourdeaut F., Cho Y.J. (2014). Medulloblastoma Down Under 2013: A report from the third annual meeting of the International Medulloblastoma Working Group. Acta Neuropathol..

[40] Cho Y.J., Tsherniak A., Tamayo P., Santagata S., Ligon A., Greulich H., Berhoukim R., Amani V., Goumnerova L., Eberhart C.G. (2011). Integrative genomic analysis of medulloblastoma identifies a molecular subgroup that drives poor clinical outcome. J. Clin. Oncol..

[41] Remke M., Hielscher T., Northcott P.A., Witt H., Ryzhova M., Wittmann A., Benner A., von Deimling A., Scheurlen W., Perry A. (2011). Adult medulloblastoma comprises three major molecular variants. J. Clin. Oncol..

[42] Kool M., Jones D.T., Jäger N., Northcott P.A., Pugh T.J., Hovestadt V., Piro R.M., Esparza L.A., Markant S.L., Remke M. (2014). Genome sequencing of SHH medulloblastoma predicts genotype-related response to smoothened inhibition. Cancer Cell.

[43] Brugières L., Remenieras A., Pierron G., Varlet P., Forget S., Byrde V., Bombled J., Puget S., Caron O., Dufour C. (2012). High frequency of germline SUFU mutations in children with desmoplastic/nodular medulloblastoma younger than 3 years of age. J. Clin. Oncol..

[44] Slade I., Murray A., Hanks S., Kumar A., Walker L., Hargrave D., Douglas J., Stiller C., Izatt L., Rahman N. (2011). Heterogeneity of familial medulloblastoma and contribution of germline PTCH1 and SUFU mutations to sporadic medulloblastoma. Fam. Cancer.

[45] Jones D.T., Jäger N., Kool M., Zichner T., Hutter B., Sultan M., Cho Y.J., Pugh T.J., Hovestadt V., Stütz A.M. (2012). Dissecting the genomic complexity underlying medulloblastoma. Nature.

[46] Pugh T.J., Weeraratne S.D., Archer T.C., Pomeranz Krummel D.A., Auclair D., Bochicchio J., Carneiro M.O., Carter S.L., Cibulskis K., Erlich R.L. (2012). Medulloblastoma exome sequencing uncovers subtype-specific somatic mutations. Nature.

[47] Cavalli F.M.G., Remke M., Rampasek L., Peacock J., Shih D.J.H., Luu B., Garzia L., Torchia J., Nor C., Morrissy A.S. (2017). Intertumoral heterogeneity within medulloblastoma subgroups. Cancer Cell.

[48] Ramaswamy V., Remke M., Bouffet E., Faria C.C., Perreault S., Cho Y.J., Shih D.J., Luu B., Dubuc A.M., Northcott P.A. (2013). Recurrence patterns across medulloblastoma subgroups: An integrated clinical and molecular analysis. Lancet Oncol..

[49] Menyhárt O., Győrffy B. (2019). Principles of tumorigenesis and emerging molecular drivers of SHH-activated medulloblastomas. Ann. Clin. Transl. Neurol..

[50] Wefers A.K., Warmuth-Metz M., Pöschl J., von Bueren A.O., Monoranu C.M., Seelos K., Peraud A., Tonn J.C., Koch A., Pietsch T. (2014). Subgroup-specific localization of human medulloblastoma based on pre-operative MRI. Acta Neuropathol..

[51] Remke M., Ramaswamy V., Peacock J., Shih D.J.H., Koelsche C., Northcott P.A., Hill N., Cavalli F.M.G., Kool M., Wang X. (2013). TERT promoter mutations are highly recurrent in SHH subgroup medulloblastoma. Acta Neuropathol..

[52] Koelsche C., Sahm F., Capper D., Reuss D., Sturm D., Jones D.T.W., Kool M., Northcott P.A., Wiestler B., Böhmer K. (2013). Distribution of TERT promoter mutations in pediatric and adult tumors of the nervous system. Acta Neuropathol..

[53] Northcott P.A., Shih D.J., Remke M., Cho Y.J., Kool M., Hawkins C., Eberhart C.G., Dubuc A., Guettouche T., Cardentey Y. (2012). Rapid, reliable, and reproducible molecular sub-grouping of clinical medulloblastoma samples. Acta Neuropathol..

[54] Goschzik T., Schwalbe E.C., Hicks D., Smith A., Zur Muehlen A., Figarella-Branger D., Doz F., Rutkowski S., Lannering B., Pietsch T. (2018). Prognostic effect of whole chromosomal aberration signatures in standard-risk, non-WNT/non-SHH medulloblastoma: A retrospective, molecular analysis of the HIT-SIOP PNET 4 trial. Lancet Oncol..

[55] Robinson G., Parker M., Kranenburg T.A., Lu C., Chen X., Ding L., Phoenix T.N., Hedlund E., Wei L., Zhu X. (2012). Novel mutations target distinct subgroups of medulloblastoma. Nature.

[56] Rudin C.M., Hann C.L., Laterra J., Hielscher T., Eberhart C.G., Mack S., Bouffet E., Clifford S.C., Hawkins C.E., French P. (2009). Treatment of medulloblastoma with hedgehog pathway inhibitor GDC-0449. N. Engl. J. Med..

[57] Northcott P.A., Dubuc A.M., Pfister S., Taylor M.D. (2012). Molecular subgroups of medulloblastoma. Expert Rev. Neurother..

[58] Forget A., Martignetti L., Puget S., Pfaff E., Shih D.J.H., Martin D.C., Castelo-Branco P., Baskin B., Ray P.N., Bouffet E. (2018). Aberrant ERBB4-SRC signaling as a hallmark of Group 4 medulloblastoma revealed by integrative phosphoproteomic profiling. Cancer Cell.

[59] Juraschka K., Taylor M.D. (2019). Medulloblastoma in the age of molecular subgroups: A review. J. Neurosurg. Pediatr..

[60] Pei Y., Moore C.E., Wang J., Tewari A.K., Eroshkin A., Cho Y.J., Witt H., Korshunov A., Read T.A., Sun J.L. (2012). An animal model of MYC-driven medulloblastoma. Cancer Cell.

[61] Northcott P.A., Shih D.J., Peacock J., Garzia L., Morrissy A.S., Zichner T., Stütz A.M., Korshunov A., Reimand J., Schumacher S.E. (2012). Subgroup-specific structural variation across 1000 medulloblastoma genomes. Nature.

[62] Swartling F.J., Grimmer M.R., Hackett C.S., Northcott P.A., Fan Q.W., Goldenberg D.D., Lau J., Masic S., Nguyen K., Yakovenko S. (2010). Pleiotropic role for MYCN in medulloblastoma. Genes Dev..

[63] Hovestadt V., Smith K.S., Bihannic L., Filbin M.G., Shaw M.L., Baumgartner A., DeWitt J.C., Groves A., Mayr L., Weisman H.R. (2019). Resolving medulloblastoma cellular architecture by single-cell genomics. Nature.

[64] Tech K., Gershom L.B., Tan A.C., Morrissy A.S., Meidinger J., Fish T., Green S.C., Liu H., Li Y., Mungall A.J. (2017). Pyruvate kinase inhibits proliferation during postnatal cerebellar neurogenesis and suppresses medulloblastoma formation. Cancer Res..

[65] Hovestadt V., Ayrault O., Swartling F.J., Robinson G.W., Pfister S.M., Northcott P.A. (2020). Medulloblastomics revisited: Biological and clinical insights from thousands of patients. Nat. Rev. Cancer.

[66] Ellison D.W., Dalton J., Kocak M., Nicholson S.L., Fraga C., Neale G., Kenney A.M., Brat D.J., Perry A., Yong W.H. (2011). Medulloblastoma: Clinicopathological correlates of SHH, WNT, and non-SHH/WNT molecular subgroups. Acta Neuropathol..

[67] Schwalbe E.C., Lindsey J.C., Nakjang S., Crosier S., Smith A.J., Hicks D., Rafiee G., Hill R.M., Iliasova A., Stone T. (2017). Novel molecular subgroups for clinical classification and outcome prediction in childhood medulloblastoma: A cohort study. Lancet Oncol..

[68] Sharma T., Schwalbe E.C., Williamson D., Sill M., Hovestadt V., Mynarek M., Rutkowski S., Robinson G.W., Gajjar A., Cavalli F. (2019). Second-generation molecular subgrouping of medulloblastoma: An international meta-analysis of Group 3 and Group 4 subtypes. Acta Neuropathol..

[69] Pomeroy S.L., Tamayo P., Gaasenbeek M., Sturla L.M., Angelo M., McLaughlin M.E., Kim J.Y., Goumnerova L.C., Black P.M., Lau C. (2002). Prediction of central nervous system embryonal tumour outcome based on gene expression. Nature.

[70] Gajjar A.J., Robinson G.W., Smith K.S., Lin T., Merchant T.E., Chintagumpala M., Mahajan A., Su J., Bouffet E., Bartels U. (2021). Outcomes by clinical and molecular features in children with medulloblastoma treated with risk-adapted therapy: Results of an international phase III trial (SJMB03). J. Clin. Oncol..

[71] von Bueren A.O., Kortmann R.D., von Hoff K., Friedrich C., Mynarek M., Müller K., Goschzik T., Zur Mühlen A., Gerber N., Warmuth-Metz M. (2016). Treatment of children and adolescents with metastatic medulloblastoma and prognostic relevance of clinical and biologic parameters. J. Clin. Oncol..

[72] Remke M., Hielscher T., Korshunov A., Northcott P.A., Bender S., Kool M., Westermann F., Benner A., Cin H., Ryzhova M. (2011). FSTL5 is a marker of poor prognosis in non-WNT/non-SHH medulloblastoma. J. Clin. Oncol..

[73] Rausch T., Jones D.T., Zapatka M., Stütz A.M., Zichner T., Weischenfeldt J., Jäger N., Remke M., Shih D., Northcott P.A. (2012). Genome sequencing of pediatric medulloblastoma links catastrophic DNA rearrangements with TP53 mutations. Cell.

[74] Skowron P., Farooq H., Cavalli F.M.G., Morrissy A.S., Ly M., Hendrikse L.D., Wang E.Y., Djambazian H., Zhu H., Mungall K.L. (2021). The transcriptional landscape of Shh medulloblastoma. Nat. Commun..

[75] Hill R.M., Kuijper S., Lindsey J.C., Petrie K., Schwalbe E.C., Barker K., Boult J.K., Williamson D., Ahmad Z., Hallsworth A. (2015). Combined MYC and P53 defects emerge at medulloblastoma relapse and define rapidly progressive, therapeutically targetable disease. Cancer Cell.

[76] Lastowska M., Al-Afghani H., Al-Balool H.H., Sheth H., Mercer E., Coxhead J.M., Redfern C.P., Peters H., Burt A.D., Santibanez-Koref M. (2013). Identification of a neuronal transcription factor network involved in medulloblastoma development. Acta Neuropathol. Commun..

[77] Rodriguez-Blanco J., Pednekar L., Penas C., Li B., Martin V., Long J., Lee E., Weiss W.A., Rodriguez C., Mehrdad N. (2017). Inhibition of WNT signaling attenuates self-renewal of SHH-subgroup medulloblastoma. Oncogene.

[78] Leary S.E., Packer R.J., Li Y., Billups C.A., Smith K.S., Jaju A., Heier L., Burger P., Walsh K., Han Y. (2021). Efficacy of carboplatin and isotretinoin in children with high-risk medulloblastoma: A randomized clinical trial from the Children’s Oncology Group. JAMA Oncol..

[79] Naeem A., Harish V., Coste S., Parasido E.M., Choudhry M.U., Kromer L.F., Ihemelandu C., Petricoin E.F., Pierobon M., Noon M.S. (2022). Regulation of Chemosensitivity in Human Medulloblastoma Cells by p53 and the PI3 Kinase Signaling Pathway. Mol. Cancer Res..

[80] Taylor M.D., Mainprize T.G., Rutka J.T. (2000). Molecular insight into medulloblastoma and central nervous system primitive neuroectodermal tumor biology from hereditary syndromes: A review. Neurosurgery.

[81] Lindsey J.C., Hill R.M., Megahed H., Lusher M.E., Schwalbe E.C., Cole M., Hogg T.L., Gilbertson R.J., Ellison D.W., Bailey S. (2011). TP53 mutations in favorable-risk Wnt/Wingless-subtype medulloblastomas. J. Clin. Oncol..

[82] Gajjar A., Pfister S.M., Taylor M.D., Gilbertson R.J. (2014). Molecular insights into pediatric brain tumors have the potential to transform therapy. Clin. Cancer Res..

[83] Pietsch T., Schmidt R., Remke M., Korshunov A., Hovestadt V., Jones D.T., Felsberg J., Kaulich K., Goschzik T., Kool M. (2014). Prognostic significance of clinical, histopathological, and molecular characteristics of medulloblastomas in the prospective HIT2000 multicenter clinical trial cohort. Acta Neuropathol..

[84] Schwalbe E.C., Williamson D., Lindsey J.C., Hamilton D., Ryan S.L., Megahed H., Garami M., Hauser P., Dembowska-Baginska B., Perek D. (2013). DNA methylation profiling of medulloblastoma allows robust subclassification and improved outcome prediction using formalin-fixed biopsies. Acta Neuropathol..

[85] Malkin D., Li F.P., Strong L.C., Fraumeni J.F., Nelson C.E., Kim D.H., Kassel J., Gryka M.A., Bischoff F.Z., Tainsky M.A. (1990). Germ line p53 mutations in a familial syndrome of breast cancer, sarcomas, and other neoplasms. Science.

[86] Tabori U., Baskin B., Shago M., Alon N., Taylor M.D., Ray P.N., Bouffet E., Malkin D., Hawkins C. (2010). Universal poor survival in children with medulloblastoma harboring somatic TP53 mutations. J. Clin. Oncol..

[87] Korshunov A., Ryzhova M., Hovestadt V., Bender S., Sturm D., Capper D., Meyer J., Schrimpf D., Kool M., Northcott P.A. (2015). Integrated analysis of pediatric glioblastoma reveals a subset of biologically favorable tumors with associated molecular prognostic markers. Acta Neuropathol..

[88] Helgager J., Pytel P., Vasudevaraja V., Lee E.Q., Snuderl M., Iorgulescu J.B., Ligon K.L. (2020). WNT-Activated Medulloblastomas with Hybrid Molecular Subtypes. JCO Precis. Oncol..

[89] Kieran M.W., Chisholm J., Casanova M., Brandes A.A., Aerts I., Bouffet E., Bailey S., Leary S., MacDonald T.J., Mechinaud F. (2017). Phase I study of oral sonidegib (LDE225) in pediatric brain and solid tumors and a phase II study in children and adults with relapsed medulloblastoma. Neuro Oncol..

[90] Robinson G.W., Kaste S.C., Chemaitilly W., Bowers D.C., Laughton S., Smith A., Gottardo N.G., Partap S., Bendel A., Wright K.D. (2017). Irreversible growth plate fusions in children with medulloblastoma treated with a targeted hedgehog pathway inhibitor. Oncotarget.

[91] Kumar V., Kumar V., McGuire T., Coulter D.W., Sharp J.G., Mahato R.I. (2017). Challenges and recent advances in medulloblastoma therapy. Trends Pharmacol. Sci..

[92] Morrissy A.S., Garzia L., Shih D.J., Zuyderduyn S., Huang X., Skowron P., Remke M., Cavalli F.M., Ramaswamy V., Lindsay P.E. (2016). Divergent clonal selection dominates medulloblastoma at recurrence. Nature.

[93] Schwalbe E.C., Lindsey J.C., Danilenko M., Hill R.M., Crosier S., Ryan S.L., Williamson D., Castle J., Hicks D., Kool M. (2025). Molecular and clinical heterogeneity within MYC-family amplified medulloblastoma is associated with survival outcomes: A multicenter cohort study. Neuro Oncol..

[94] Robinson G.W., Rudneva V.A., Buchhalter I., Billups C.A., Waszak S.M., Smith K.S., Bowers D.C., Bendel A., Fisher P.G., Partap S. (2018). Risk-adapted therapy for young children with medulloblastoma (SJYC07): Therapeutic and molecular outcomes from a multicentre, phase 2 trial. Lancet Oncol..

[95] Ryan S.L., Schwalbe E.C., Cole M., Lu Y., Lusher M.E., Megahed H., O’Toole K., Nicholson S.L., Bognar L., Garami M. (2012). MYC family amplification and clinical risk-factors interact to predict an extremely poor prognosis in childhood medulloblastoma. Acta Neuropathol..

[96] Zitterbart K., Filkova H., Tomasikova L., Necesalova E., Zambo I., Kantorova D., Slamova I., Vranova V., Zezulkova D., Pesakova M. (2011). Low-level copy number changes of MYC genes have a prognostic impact in medulloblastoma. J. Neuro Oncol..

[97] Bandopadhayay P., Bergthold G., Nguyen B., Schubert S., Gholamin S., Tang Y., Bolin S., Schumacher S.E., Zeid R., Masoud S. (2014). BET bromodomain inhibition of MYC-amplified medulloblastoma. Clin. Cancer Res..

[98] Williamson D., Schwalbe E.C., Hicks D., Aldinger K.A., Lindsey J.C., Crosier S., Richardson S., Goddard J., Hill R.M., Castle J. (2019). Medulloblastoma group 3 and 4 tumors comprise a clinically and biologically significant expression continuum reflecting human cerebellar development. Cell Rep..

[99] Adhvaryu V.V., Gurav M., Deshpande G., Rumde R., Shetty O., Sahay A., Sahu A., Dasgupta A., Chatterji A., Gupta T. (2025). Adult Medulloblastoma: Clinicomolecular Spectrum, An Institutional Experience. Int. J. Surg. Pathol..

[100] Tamayo P., Cho Y.J., Tsherniak A., Greulich H., Ambrogio L., Schouten-van Meeteren N., Zhou T., Buxton A., Kool M., Meyerson M. (2011). Predicting relapse in patients with medulloblastoma by integrating evidence from clinical and genomic features. J. Clin. Oncol..

[101] Mizushima M., Okamoto M., Yamaguchi S., Oki S., Motegi H., Sugiyama M., Manabe A., Shimizu A., Nishioka K., Hashimoto T. (2023). Slow-growing WNT medulloblastoma with atypical magnetic resonance imaging findings: Illustrative case. J. Neurosurg. Case Lessons.

[102] Goschzik T., Zur Muehlen A., Doerner E., Waha A., Friedrich C., Hau P., Pietsch T. (2021). Medulloblastoma in Adults: Cytogenetic Phenotypes Identify Prognostic Subgroups. J. Neuropathol. Exp. Neurol..

[103] Korshunov A., Remke M., Werft W., Benner A., Ryzhova M., Witt H., Sturm D., Wittmann A., Schöttler A., Felsberg J. (2010). Adult and pediatric medulloblastomas are genetically distinct and require different algorithms for molecular risk stratification. J. Clin. Oncol..

[104] Schmidt C., Cohen S., Gudenas B.L., Husain S., Carlson A., Westelman S., Wang L., Phillips J.J., Northcott P.A., Weiss W.A. (2024). PRDM6 promotes medulloblastoma by repressing chromatin accessibility and altering gene expression. Sci. Rep..

[105] Venneti S., Garimella M.T., Sullivan L.M., Martinez D., Huse J.T., Heguy A., Santi M., Thompson C.B., Judkins A.R. (2013). Evaluation of histone 3 lysine 27 trimethylation (H3K27me3) and enhancer of Zest 2 (EZH2) in pediatric glial and glioneuronal tumors shows decreased H3K27me3 in H3F3A K27M mutant glioblastomas. Brain Pathol..

[106] Pei Y., Liu K.W., Wang J., Garancher A., Tao R., Esparza L.A., Maier D.L., Udaka Y.T., Murad N., Morrissy S. (2016). HDAC and PI3K antagonists cooperate to inhibit growth of MYC-driven medulloblastoma. Cancer Cell.

[107] Tech K., Deshmukh M., Gershon T.R. (2015). Adaptations of energy metabolism during cerebellar neurogenesis are co-opted in medulloblastoma. Cancer Lett..

[108] Sursal T., Ronecker J.S., Dicpinigaitis A.J., Mohan A.L., Tobias M.E., Gandhi C.D., Jhanwar-Uniyal M. (2022). Molecular Stratification of Medulloblastoma: Clinical Outcomes and Therapeutic Interventions. Anticancer. Res..

[109] Zeltzer P.M., Boyett J.M., Finlay J.L., Albright A.L., Rorke L.B., Milstein J.M., Allen J.C., Stevens K.R., Stanley P., Li H. (1999). Metastasis stage, adjuvant treatment, and residual tumor are prognostic factors for medulloblastoma in children: Conclusions from the Children’s Cancer Group 921 randomized phase III study. J. Clin. Oncol..

[110] Killela P.J., Reitman Z.J., Jiao Y., Bettegowda C., Agrawal N., Diaz L.A., Friedman A.H., Friedman H., Gallia G.L., Giovanella B.C. (2013). TERT promoter mutations occur frequently in gliomas and a subset of tumors derived from cells with low rates of self-renewal. Proc. Natl. Acad. Sci. USA.

[111] Terzi N.K., Yilmaz I., Oz A.B. (2022). The Place and Prognostic Value of TERT Promoter Mutation in Molecular Classification in Grade II-III Glial Tumors and Primary Glioblastomas. Turk. Patoloji Derg..

[112] Theil A.F., Hoeijmakers J.H. (2025). Expanding the landscape of nucleotide excision repair disorders: From discovery to therapy. J. Clin. Investig..

[113] Bouffet E., Larouche V., Campbell B.B., Merico D., de Borja R., Aronson M., Durno C., Krueger J., Cabric V., Ramaswamy V. (2016). Immune checkpoint inhibition for hypermutant glioblastoma multiforme resulting from germline biallelic mismatch repair deficiency. J. Clin. Oncol..

[114] Muñoz Perez N., Pensabene J.M., Galbo P.M., Sadeghipour N., Xiu J., Moziak K., Yazejian R.M., Welch R.L., Bell W.R., Sengupta S. (2024). VISTA Emerges as a Promising Target against Immune Evasion Mechanisms in Medulloblastoma. Cancers.

[115] Zou H., Poore B., Broniscer A., Pollack I.F., Hu B. (2020). Molecular Heterogeneity and Cellular Diversity: Implications for Precision Treatment in Medulloblastoma. Cancers.

[116] Zhang Z.W., Teng X., Zhao F., Ma C., Zhang J., Xiao L.F., Wang Y., Chang M., Tian Y., Li C. (2022). METTL3 regulates m6A methylation of PTCH1 and GLI2 in Sonic hedgehog signaling to promote tumor progression in SHH-medulloblastoma. Cell Rep..

[117] Albright A.L., Wisoff J.H., Zeltzer P.M., Boyett J., Rorke L.B., Stanley P., Geyer J.R., Milstein J.M. (1995). Prognostic factors in children with supratentorial (nonpineal) primitive neuroectodermal tumors. A neurosurgical perspective from the Children’s Cancer Group. Pediatr. Neurosurg..

[118] Schmitz A.K., Munoz-Bendix C., Remke M., Brozou T., Borkhardt A., Hänggi D., Beez T. (2022). Second-look surgery after pediatric brain tumor resection—Single center analysis of morbidity and volumetric efficacy. Brain Spine.

[119] Neth B.J., Raghunathan A., Kizilbash S.H., Uhm J.H., Breen W.G., Johnson D.R., Daniels D.J., Sener U., Carabenciov I.D., Campian J.L. (2023). Management and Long-term Outcomes of Adults with Medulloblastoma: A Single-Center Experience. Neurology.

[120] Robertson P.L., Muraszko K.M., Holmes E.J., Sposto R., Packer R.J., Gajjar A., Dias M.S., Allen J.C. (2006). Incidence and severity of postoperative cerebellar mutism syndrome in children with medulloblastoma: A prospective study by the Children’s Oncology Group. J. Neurosurg..

[121] Sala F., Lanteri P., Bricolo A. (2004). Motor evoked potential monitoring for spinal cord tumor surgery. Adv. Tech. Stand. Neurosurg..

[122] Kulkarni A.V., Drake J.M., Lamberti-Pasculli M. (2001). Cerebrospinal fluid shunt infection: A prospective study of risk factors. J. Neurosurg..

[123] Strother D., Lafay-Cousin L. (2021). Adjuvant therapy for high-risk medulloblastoma: More is better?. Neuro Oncol..

[124] Tarbell N.J., Friedman H., Polkinghorn W.R., Yock T., Zhou T., Chen Z., Burger P., Barnes P., Kun L. (2013). High-risk medulloblastoma: A pediatric oncology group randomized trial of chemotherapy before or after radiation therapy (POG 9031). J. Clin. Oncol..

[125] Michalski J.M., Janss A.J., Vezina L.G., Smith K.S., Billups C.A., Burger P.C., Embry L.M., Cullen P.L., Hardy K.K., Pomeroy S.L. (2021). Children’s Oncology Group Phase III Trial of Reduced-Dose and Reduced-Volume Radiotherapy with Chemotherapy for Newly Diagnosed Average-Risk Medulloblastoma. J. Clin. Oncol..

[126] Yock T.I., Yeap B.Y., Ebb D.H., Weyman E., Eaton B.R., Sherry N.A., Jones R.M., MacDonald S.M., Pulsifer M.B., Lavally B. (2016). Long-term toxic effects of proton radiotherapy for paediatric medulloblastoma: A phase 2 single-arm study. Lancet Oncol..

[127] Brown A.P., Barney C.L., Grosshans D.R., McAleer M.F., de Groot J.F., Puduvalli V.K., Tucker S.L., Crawford C.N., Khan M., Khatua S. (2013). Proton beam craniospinal irradiation reduces acute toxicity for adults with medulloblastoma. Int. J. Radiat. Oncol. Biol. Phys..

[128] Ang C., Hauerstock D., Guiot M.C., Kasymjanova G., Roberge D., Kavan P., Muanza T. (2008). Characteristics and outcomes of medulloblastoma in adults. Pediatr. Blood Cancer.

[129] Chan A.W., Tarbell N.J., Black P.M., Louis D.N., Frosch M.P., Ancukiewicz M., Chapman P., Loeffler J.S. (2000). Adult medulloblastoma: Prognostic factors and patterns of relapse. Neurosurgery.

[130] Harrabi S.B., Bougatf N., Mohr A., Haberer T., Herfarth K., Combs S.E., Debus J., Adeberg S. (2016). Dosimetric advantages of proton therapy over conventional radiotherapy with photons in young patients and adults with low-grade glioma. Strahlenther. Onkol..

[131] Majd N., Penas-Prado M. (2019). Updates on Management of Adult Medulloblastoma. Curr. Treat. Options Oncol..

[132] Kann B.H., Park H.S., Liang Y., Yeboa D.N., Kelly J.R., Baehring J.M., Becker K.P., Yu J.B., Bindra R.S., Roberts K.B. (2017). Adjuvant chemotherapy and overall survival in adult medulloblastoma. Neuro Oncol..

[133] Packer R.J., Goldwein J., Nicholson H.S., Vezina L.G., Allen J.C., Ris M.D., Muraszko K., Rorke L.B., Wara W.M., Cohen B.H. (1999). Treatment of children with medulloblastomas with reduced-dose craniospinal radiation therapy and adjuvant chemotherapy: A Children’s Cancer Group Study. J. Clin. Oncol..

[134] Greenberg H.S., Chamberlain M.C., Glantz M.J., Wang S. (2001). Adult medulloblastoma: Multiagent chemotherapy. Neuro Oncol..

[135] Dunkel I.J., Finlay J.L., Boyett J.M., Rosenblum M., Garvin J.H., Bostrom B.C., Goldman S., Sender L.S., Gardner S.L., Li H. (1998). High-dose carboplatin, thiotepa, and etoposide with autologous stem-cell rescue for patients with recurrent medulloblastoma. Children’s Cancer Group. J. Clin. Oncol..

[136] Ruggiero A., Talloa D., Romano A., Attinà G., Mastrangelo S., Maurizi P., Verdolotti T., Tamburrini G., Chiesa S., di Bonaventura R. (2025). Outcomes and Toxicity of Adult Medulloblastoma Treated with Pediatric Multimodal Protocols: A Single-Institution Experience. Oncol. Res..

[137] Brandes A.A., Paris M.K. (2004). Review of the prognostic factors in medulloblastoma of children and adults. Crit. Rev. Oncol. Hematol..

[138] Friedrich C., von Bueren A.O., von Hoff K., Kwiecien R., Pietsch T., Warmuth-Metz M., Hau P., Deinlein F., Kuehl J., Kortmann R.D. (2013). Treatment of adult nonmetastatic medulloblastoma patients according to the paediatric HIT 2000 protocol: A prospective observational multicentre study. Eur. J. Cancer.

[139] Yauch R.L., Dijkgraaf G.J., Alicke B., Januario T., Ahn C.P., Holcomb T., Pujara K., Stinson J., Callahan C.A., Tang T. (2009). Smoothened mutation confers resistance to a Hedgehog pathway inhibitor in medulloblastoma. Science.

[140] Liu X., Zhang Y., Li Y., Wang J., Ding H., Huang W., Ding C., Liu H., Tan W., Zhang A. (2021). Development of hedgehog pathway inhibitors by epigenetically targeting GLI through BET bromodomain for the treatment of medulloblastoma. Acta Pharm. Sin. B.

[141] Villani A., Fabbrocini G., Costa C., Ocampo-Garza S.S., Lallas A., Scalvenzi M. (2021). Expert opinion on sonidegib efficacy, safety and tolerability. Expert. Opin. Drug Saf..

[142] Pak E., MacKenzie E.L., Zhao X., Pazyra-Murphy M.F., Park P.M.C., Wu L., Shaw D.L., Addleson E.C., Cayer S.S., Lopez B.G. (2019). A large-scale drug screen identifies selective inhibitors of class I HDACs as a potential therapeutic option for SHH medulloblastoma. Neuro Oncol..

[143] Sasaki H., Hui C., Nakafuku M., Kondoh H. (1997). A binding site for Gli proteins is essential for HNF-3beta floor plate enhancer activity in transgenics and can respond to Shh in vitro. Development.

[144] Marquardt V., Theruvath J., Pauck D., Picard D., Qin N., Blümel L., Maue M., Bartl J., Ahmadov U., Langini M. (2023). Tacedinaline (CI-994), a class I HDAC inhibitor, targets intrinsic tumor growth and leptomeningeal dissemination in MYC-driven medulloblastoma while making them susceptible to anti-CD47-induced macrophage phagocytosis via NF-kB-TGM2 driven tumor inflammation. J. Immunother. Cancer.

[145] Kling M.J., Kesherwani V., Mishra N.K., Alexander G., McIntyre E.M., Ray S., Challagundla K.B., Joshi S.S., Coulter D.W., Chaturvedi N.K. (2022). A novel dual epigenetic approach targeting BET proteins and HDACs in Group 3 (MYC-driven) Medulloblastoma. J. Exp. Clin. Cancer Res..

[146] Veo B., Danis E., Pierce A., Sola I., Wang D., Foreman N.K., Jin J., Ma A., Serkova N., Venkataraman S. (2019). Combined functional genomic and chemical screens identify SETD8 as a therapeutic target in MYC-driven medulloblastoma. JCI Insight.

[147] Smoll N.R. (2012). Relative survival of childhood and adult medulloblastomas and primitive neuroectodermal tumors (PNETs). Cancer.

[148] Lai R. (2008). Survival of patients with adult medulloblastoma: A population-based study. Cancer.

[149] Li Q., Dai Z., Cao Y., Wang L. (2018). Comparing children and adults with medulloblastoma: A SEER based analysis. Oncotarget.

[150] Padovani L., Sunyach M.P., Perol D., Mercier C., Alapetite C., Haie-Meder C., Hoffstetter S., Muracciole X., Kerr C., Wagner J.P. (2007). Common strategy for adult and pediatric medulloblastoma: A multicenter series of 253 adults. Int. J. Radiat. Oncol. Biol. Phys..

[151] Riffaud L., Saikali S., Leray E., Hamlat A., Haegelen C., Vauleon E., Lesimple T. (2009). Survival and prognostic factors in a series of adults with medulloblastomas. J. Neurosurg..

[152] Sabel M., Fleischhack G., Tippelt S., Gustafsson G., Doz F., Kortmann R., Massimino M., Navajas A., von Hoff K., Rutkowski S. (2016). Relapse patterns and outcome after relapse in standard risk medulloblastoma: A report from the HIT-SIOP-PNET4 study. J. Neuro Oncol..

[153] Hill R.M., Richardson S., Schwalbe E.C., Hicks D., Lindsey J.C., Crosier S., Rafiee G., Grabovska Y., Wharton S.B., Jacques T.S. (2020). Time, pattern, and outcome of medulloblastoma relapse and their association with tumour biology at diagnosis and therapy: A multicentre cohort study. Lancet Child. Adolesc. Health.

[154] Massimino M., Biassoni V., Gandola L., Garrè M.L., Gatta G., Giangaspero F., Poggi G., Rutkowski S. (2016). Childhood medulloblastoma. Crit. Rev. Oncol. Hematol..

[155] Grundy R.G., Wilne S.H., Robinson K.J., Ironside J.W., Cox T., Chong W.K., Michalski A., Campbell R.H., Bailey C.C., Thorp N. (2010). Primary postoperative chemotherapy without radiotherapy for treatment of brain tumours other than ependymoma in children under 3 years: Results of the first UKCCSG/SIOP CNS 9204 trial. Eur. J. Cancer.

[156] Wetmore C., Herington D., Lin T., Onar-Thomas A., Gajjar A., Merchant T.E. (2014). Reirradiation of recurrent medulloblastoma: Does clinical benefit outweigh cumulative toxicity?. Cancer.

[157] Cheuk D.K., Lee T.L., Chiang A.K., Ha S.Y., Chan G.C. (2008). Autologous hematopoietic stem cell transplantation for high-risk brain tumors in children. J. Neuro Oncol..

[158] Cefalo M.G., Maurizi P., Arlotta A., Scalzone M., Attinà G., Ruggiero A., Riccardi R. (2010). Hepatic veno-occlusive disease: A chemotherapy-related toxicity in children with malignancies. Paediatr. Drugs.

[159] Romano A., Triarico S., Rinninella E., Natale L., Brizi M.G., Cintoni M., Raoul P., Maurizi P., Attinà G., Mastrangelo S. (2022). Clinical Impact of Nutritional Status and Sarcopenia in Pediatric Patients with Bone and Soft Tissue Sarcomas: A Pilot Retrospective Study (SarcoPed). Nutrients.

[160] Palmer S.L., Goloubeva O., Reddick W.E., Glass J.O., Gajjar A., Kun L., Merchant T.E., Mulhern R. (2001). Patterns of intellectual development among survivors of pediatric medulloblastoma: A longitudinal analysis. J. Clin. Oncol..

[161] Mabbott D.J., Spiegler B.J., Greenberg M.L., Rutka J.T., Hyder D.J., Bouffet E. (2005). Serial evaluation of academic and behavioral outcome after treatment with cranial radiation in childhood. J. Clin. Oncol..

[162] Ellenberg L., Liu Q., Gioia G., Yasui Y., Packer R.J., Mertens A., Donaldson S.S., Stovall M., Kadan-Lottick N., Armstrong G. (2009). Neurocognitive status in long-term survivors of childhood CNS malignancies: A report from the Childhood Cancer Survivor Study. Neuropsychology.

[163] Gurney J.G., Krull K.R., Kadan-Lottick N., Nicholson H.S., Nathan P.C., Zebrack B., Tersak J.M., Ness K.K. (2009). Social outcomes in the Childhood Cancer Survivor Study cohort. J. Clin. Oncol..

[164] Chemaitilly W., Li Z., Huang S., Ness K.K., Clark K.L., Green D.M., Barnes N., Armstrong G.T., Krasin M.J., Srivastava D.K. (2015). Anterior hypopituitarism in adult survivors of childhood cancers treated with cranial radiotherapy: A report from the St Jude Lifetime Cohort study. J. Clin. Oncol..

[165] Darzy K.H., Shalet S.M. (2009). Hypopituitarism following radiotherapy. Pituitary.

[166] van Iersel L., Li Z., Srivastava D.K., Brinkman T.M., Bjornard K.L., Wilson C.L., Green D.M., Merchant T.E., Pui C.H., Howell R.M. (2019). Hypothalamic-pituitary disorders in childhood cancer survivors: Prevalence, risk factors and long-term health outcomes. J. Clin. Endocrinol. Metab..

[167] Gurney J.G., Tersak J.M., Ness K.K., Landier W., Matthay K.K., Schmidt M.L. (2007). Hearing loss, quality of life, and academic problems in long-term neuroblastoma survivors: A report from the Children’s Oncology Group. Pediatrics.

[168] Rivetti S., Romano A., Mastrangelo S., Attinà G., Maurizi P., Ruggiero A. (2023). Aminoglycosides-Related Ototoxicity: Mechanisms, Risk Factors, and Prevention in Pediatric Patients. Pharmaceuticals.

[169] Friedman D.L., Whitton J., Leisenring W., Mertens A.C., Hammond S., Stovall M., Donaldson S.S., Meadows A.T., Robison L.L., Neglia J.P. (2010). Subsequent neoplasms in 5-year survivors of childhood cancer: The Childhood Cancer Survivor Study. J. Natl. Cancer Inst..

[170] Neglia J.P., Robison L.L., Stovall M., Liu Y., Packer R.J., Hammond S., Yasui Y., Kasper C.E., Mertens A.C., Donaldson S.S. (2006). New primary neoplasms of the central nervous system in survivors of childhood cancer: A report from the Childhood Cancer Survivor Study. J. Natl. Cancer Inst..

[171] Hisada M., Garber J.E., Fung C.Y., Fraumeni J.F., Li F.P. (1998). Multiple primary cancers in families with Li-Fraumeni syndrome. J. Natl. Cancer Inst..

[172] Zebrack B.J., Gurney J.G., Oeffinger K., Whitton J., Packer R.J., Mertens A., Turk N., Castleberry R., Dreyer Z., Robison L.L. (2004). Psychological outcomes in long-term survivors of childhood brain cancer: A report from the childhood cancer survivor study. J. Clin. Oncol..

[173] Maddrey A.M., Bergeron J.A., Lombardo E.R., McDonald N.K., Mulne A.F., Barenberg P.D., Bowers D.C. (2005). Neuropsychological performance and quality of life of 10 year survivors of childhood medulloblastoma. J. Neuro Oncol..

[174] Wyns C., Collienne C., Shenfield F., Robert A., Laurent P., Roegiers L., Brichard B. (2015). Fertility preservation in the male pediatric population: Factors influencing the decision of parents and children. Hum. Reprod..

[175] Loren A.W., Mangu P.B., Beck L.N., Brennan L., Magdalinski A.J., Partridge A.H., Quinn G., Wallace W.H., Oktay K. (2013). Fertility preservation for patients with cancer: American Society of Clinical Oncology clinical practice guideline update. J. Clin. Oncol..

[176] Hedberg J., Studebaker A., Smith L., Chen C.Y., Westfall J.J., Cam M., Gross A., Hernandez-Aguirre I., Martin A., Kim D. (2023). Oncolytic virus-driven immune remodeling revealed in mouse medulloblastomas at single cell resolution. Mol. Ther. Oncolytics.

[177] Smith S.M.C., Bianski B.M., Orr B.A., Harknett G., Onar-Thomas A., Gilbertson R.J., Merchant T.E., Roussel M.F., Tinkle C.L. (2019). Preclinical Modeling of Image-Guided Craniospinal Irradiation for Very-High-Risk Medulloblastoma. Int. J. Radiat. Oncol. Biol. Phys..

[178] Huang T.Y., Piunti A., Lulla R.R., Qi J., Horbinski C.M., Tomita T., James C.D., Shilatifard A., Saratsis A.M. (2017). Detection of Histone H3 mutations in cerebrospinal fluid-derived tumor DNA from children with diffuse midline glioma. Acta Neuropathol. Commun..

[179] Escudero L., Llort A., Arias A., Diaz-Navarro A., Martínez-Ricarte F., Rubio-Perez C., Mayor R., Caratù G., Martínez-Sáez E., Vázquez-Méndez É. (2020). Circulating tumour DNA from the cerebrospinal fluid allows the characterisation and monitoring of medulloblastoma. Nat. Commun..

[180] Stallard S., Savelieff M.G., Wierzbicki K., Mullan B., Miklja Z., Bruzek A., Garcia T., Siada R., Anderson B., Singer B.H. (2018). CSF H3F3A K27M circulating tumor DNA copy number quantifies tumor growth and in vitro treatment response. Acta Neuropathol. Commun..

[181] Pham C.D., Flores C., Yang C., Pinheiro E.M., Yearley J.H., Sayour E.J., Pei Y., Moore C., McLendon R.E., Huang J. (2016). Differential immune microenvironments and response to immune checkpoint blockade among molecular subtypes of murine medulloblastoma. Clin. Cancer Res..

[182] Majzner R.G., Heitzeneder S., Mackall C.L. (2017). Harnessing the immunotherapy revolution for the treatment of childhood cancers. Cancer Cell.

[183] Majzner R.G., Theruvath J.L., Nellan A., Heitzeneder S., Cui Y., Mount C.W., Rietberg S.P., Linde M.H., Xu P., Rota C. (2019). CAR T cells targeting B7-H3, a pan-cancer antigen, demonstrate potent preclinical activity against pediatric solid tumors and brain tumors. Clin. Cancer Res..

[184] Boulay G., Sandoval G.J., Riggi N., Iyer S., Buisson R., Naigles B., Awad M.E., Rengarajan S., Volorio A., McBride M.J. (2017). Cancer-specific retargeting of BAF complexes by a prion-like domain. Cell.

[185] Otto T., Horn S., Brockmann M., Eilers U., Schüttrumpf L., Popov N., Kenney A.M., Schulte J.H., Beijersbergen R., Christiansen H. (2009). Stabilization of N-Myc is a critical function of Aurora A in human neuroblastoma. Cancer Cell.

[186] Perrone M.G., Ruggiero A., Centonze A., Carrieri A., Ferorelli S., Scilimati A. (2021). Diffuse Intrinsic Pontine Glioma (DIPG): Breakthrough and Clinical Perspective. Curr. Med. Chem..

[187] Dey A., Robitaille M., Remke M., Maier C., Malhotra A., Gregorieff A., Wrana J.L., Taylor M.D., Angers S., Kenney A.M. (2016). YB-1 is elevated in medulloblastoma and drives proliferation in Sonic hedgehog-dependent cerebellar granule neuron progenitor cells and medulloblastoma cells. Oncogene.

[188] Simbilyabo L.Z., Yang L., Wen J., Liu Z. (2024). The unfolded protein response machinery in glioblastoma genesis, chemoresistance and as a druggable target. CNS Neurosci. Ther..

[189] Bleyer A., Budd T., Montello M. (2006). Adolescents and young adults with cancer: The scope of the problem and criticality of clinical trials. Cancer.

[190] Fern L.A., Whelan J.S. (2010). Recruitment of adolescents and young adults to cancer clinical trials—International comparisons, barriers, and implications. Semin. Oncol..

[191] Ramaswamy V., Taylor M.D. (2017). Medulloblastoma: From Myth to Molecular. J. Clin. Oncol..

[192] Liu Z., Ren S., Zhang H., Liao Z., Liu Z., An X., Cheng J., Li C., Gong J., Niu H. (2025). Multiparametric MRI-based machine learning system of molecular subgroups and prognosis in medulloblastoma. Eur. Radiol..

[193] Capper D., Jones D.T.W., Sill M., Hovestadt V., Schrimpf D., Sturm D., Koelsche C., Sahm F., Chavez L., Reuss D.E. (2018). DNA methylation-based classification of central nervous system tumours. Nature.

[194] Louis D.N., Perry A., Reifenberger G., von Deimling A., Figarella-Branger D., Cavenee W.K., Ohgaki H., Wiestler O.D., Kleihues P., Ellison D.W. (2016). The 2016 World Health Organization Classification of Tumors of the Central Nervous System: A summary. Acta Neuropathol..

[195] Ramírez-Chiquito J.C., Juárez-Méndez S. (2025). Strategies for the Molecular Classification of Medulloblastoma. Biomedicines.

